# Causal inference for long-term survival in randomised trials with treatment switching: Should re-censoring be applied when estimating counterfactual survival times?

**DOI:** 10.1177/0962280218780856

**Published:** 2018-06-25

**Authors:** NR Latimer, IR White, KR Abrams, U Siebert

**Affiliations:** 1 School of Health and Related Research, University of Sheffield, Sheffield, UK; 2 MRC Clinical Trials Unit, University College London, London, UK; 3 Department of Health Sciences, University of Leicester, Leicester, UK; 4 Department of Public Health, Health Services Research and Health Technology Assessment, UMIT – University for Health Sciences, Medical Informatics and Technology, Hall i.T., Austria; 5 Oncotyrol – Center for Personalized Cancer Medicine, Innsbruck, Austria; 6 Harvard T.H. Chan School of Public Health and Massachusetts General Hospital, Harvard Medical School, Boston, MA, USA

**Keywords:** Treatment switching, treatment crossover, survival analysis, overall survival, oncology, health technology assessment, time-to-event outcomes, prediction, re-censoring

## Abstract

Treatment switching often has a crucial impact on estimates of effectiveness and cost-effectiveness of new oncology treatments. Rank preserving structural failure time models (RPSFTM) and two-stage estimation (TSE) methods estimate ‘counterfactual’ (i.e. had there been no switching) survival times and incorporate re-censoring to guard against informative censoring in the counterfactual dataset. However, re-censoring causes a loss of longer term survival information which is problematic when estimates of long-term survival effects are required, as is often the case for health technology assessment decision making. We present a simulation study designed to investigate applications of the RPSFTM and TSE with and without re-censoring, to determine whether re-censoring should always be recommended within adjustment analyses. We investigate a context where switching is from the control group onto the experimental treatment in scenarios with varying switch proportions, treatment effect sizes, treatment effect changes over time, survival function shapes, disease severity and switcher prognosis. Methods were assessed according to their estimation of control group restricted mean survival that would be observed in the absence of switching, up to the end of trial follow-up. We found that analyses which re-censored usually produced negative bias (i.e. underestimating control group restricted mean survival and overestimating the treatment effect), whereas analyses that did not re-censor consistently produced positive bias which was often smaller in magnitude than the bias associated with re-censored analyses, particularly when the treatment effect was high and the switching proportion was low. The RPSFTM with re-censoring generally resulted in increased bias compared to the other methods. We believe that analyses should be conducted with and without re-censoring, as this may provide decision-makers with useful information on where the true treatment effect is likely to lie. Incorporating re-censoring should not always represent the default approach when the objective is to estimate long-term survival times and treatment effects.

## 1 Introduction

Treatment switching commonly occurs in randomised controlled trials (RCTs), whereby patients randomised to the control group are permitted to switch onto the experimental treatment during trial follow-up. Switching is permitted primarily due to ethical considerations, and the rationale for switching, its implications and analytical methods for adjusting for it have been the focus of much discussion in the literature.^1–4^ Switching in trials is likely to continue to occur and can have a large impact on estimates of the effectiveness of new treatments if treatment is efficacious and no adjustments for switching are made. It is therefore important for regulators and health technology assessors to engage with methods that attempt to adjust for switching. Several statistical adjustment methods are available, but all make strong assumptions that are not possible to test perfectly. In addition, each of these methods can be applied in a multitude of ways and seemingly innocuous choices around how a particular method is applied can importantly affect the results they produce. This is sure to influence the thinking of decision makers when they seek to interpret the results of adjustment analyses, and may lead to a lack of trust in adjustment methods. It has been suggested that decision makers require manufacturers to describe and justify adjustment analyses in detail – including the rationale for each application decision made – in order that robust and informed decisions can be made.^5,6^

Methods that have most commonly been used to adjust for treatment switching are Rank Preserving Structural Failure Time Models (RPSFTM), two-stage estimation, and inverse probability of censoring weighting (IPCW).^4,5^ RPSFTM and two-stage estimation involve the estimation of counterfactual survival times – that is, survival times that would have been observed if treatment switching had not occurred. The use of counterfactual survival times is problematic when censoring is present, and Robins and colleagues originally proposed re-censoring – which will be described in the next section – as a solution.^7,8^ Branson and Whitehead subsequently suggested that re-censoring was not required,^9^ but White responded to this by performing a simulation study in support of re-censoring.^10^

Whether or not to apply re-censoring represents an application decision that can have a substantial impact on the results of RPSFTM and two-stage estimation analyses. In a recently published study, Latimer et al. presented a series of adjustment analyses applied to a trial analysing the effect of trametinib compared to chemotherapy in patients with metastatic melanoma.^11^ A standard intention-to-treat (ITT) analysis resulted in a hazard ratio (HR) of 0.72 (95% confidence interval (CI) 0.52–0.98). However, 67% of control group patients had switched onto the experimental treatment. An RPSFTM analysis designed to adjust for the treatment switching gave a HR of 0.38 (95% CI 0.15–0.95) when re-censoring was applied, and an HR of 0.49 (95% CI 0.25–0.96) when re-censoring was not applied. The HRs for a two-stage analysis to adjust for the treatment switching were 0.43 (95% CI 0.20–0.96) with re-censoring and 0.53 (95% CI 0.29–0.97) without re-censoring. Such substantial differences in the point-estimate of the treatment effect can be critical particularly for estimates of the expected cost-effectiveness of new interventions – overall survival benefit estimates are often the most influential parameters within cost-effectiveness models of cancer interventions.^12^ Cost-effectiveness analyses are key factors in reimbursement decisions made on new healthcare interventions around the world.^13–16^

It is generally recommended to apply re-censoring when using RPSFTM and two-stage estimation methods.^7,8,10,17^ However, it is recognised that whilst re-censoring helps avoid one type of bias – informative censoring – it can result in a type of missing information bias when the treatment effect changes over time, or when long-term trends in hazards are not established in the short term, because longer-term information is lost.^1,17–20^ It may therefore be possible that in some situations analyses which do not re-censor are preferable to analyses which do. Currently, little is known about the impact of re-censoring in realistic scenarios, or how results should be interpreted when the choice of whether or not to re-censor has a large impact on the estimated treatment effect. Simulation studies have shown that adjustment methods produce varying levels of bias depending upon factors such as the switch proportion and the treatment effect size, but have only considered applications of adjustment methods that include re-censoring.^21–23^ In this paper, we conduct a new simulation study to investigate the performance of adjustment methods with and without applying re-censoring. Our objective is to determine whether it is possible to discern the likely impact of re-censoring in various scenarios, in order that expectations over the likely bias associated with analyses that do or do not re-censor can be informed. This should allow analysts and decision-makers to better interpret the results of adjustment analyses, enabling more constructive use of adjustment methods.

## 2 Methods

### 2.1 Statistical adjustment methods

The RPSFTM^24^ and two-stage estimation methods^22^ can be used to estimate counterfactual survival times in the presence of treatment switching in RCTs.

The simple one-parameter version of the RPSFTM splits the observed event time, *T_i_*, for each patient into time spent on the control treatment, $T_{A_{i}}$ , and time spent on the intervention treatment, $T_{B_{i}}$ . For patients who are randomised to the intervention treatment, and who do not switch onto the control treatment, $T_{A_{i}}$ is equal to zero. For patients randomised to the control group who do not switch onto the intervention, $T_{B_{i}}$ is equal to zero. However, for patients who switch treatments, both $T_{A_{i}}$ and $T_{B_{i}}$ will be greater than zero. The RPSFTM method relates *T_i_* to the counterfactual survival time (*U_i_*) with the following causal model

(1) $$U_{i}=T_{A_{i}}+e^{\psi }T_{B_{i}}$$

Here $e^{-\psi }$ represents the acceleration factor (AF) associated with the intervention – the factor by which treatment increases an individual’s expected survival time. The RPSFTM assumes that there is a common treatment effect associated with the experimental treatment (i.e. that the treatment effect, $e^{\psi }$ , is the same no matter when the treatment is received) and that if no patients received the experimental treatment average survival times in the randomised groups would be equal. Given these assumptions, g-estimation is used to estimate ψ, with the true value being that for which counterfactual survival times (*U_i_*) are independent of randomised group.^24^ This is done by computing *U_i_* for a range of values of ψ and each time testing whether the *U_i_* are independent of randomised group.

The two-stage estimation method also involves estimating counterfactual survival times. The counterfactual survival model (equation (1)) is again used, but the two-stage estimation method estimates ψ based upon an assumption of no unmeasured confounding. Under the simple two-stage method, it is assumed that treatment switching only occurs after a disease-related secondary baseline, such as disease progression. Then (assuming switching is only from the control group onto the experimental treatment), post-secondary baseline survival times in control group patients who switch onto the experimental treatment are compared to those in control group patients who do not switch, using a parametric accelerated failure time model (e.g. Weibull or Generalised Gamma), controlling for prognostic covariates measured at the secondary baseline time-point and including the switch indicator as a time-dependent variable which equals ‘1’ after the time of switch. A treatment effect (ψ) associated with switching is then obtained, and is incorporated into (equation (1)) to estimate counterfactual survival times in switching patients. Therefore, whilst the RPSFTM uses the randomisation and common treatment effect assumptions combined with g-estimation to account for potential prognostic differences between switchers and non-switchers, the two-stage method accounts for these differences by including important prognostic factors in the model used to estimate ψ.

Censoring is problematic for the RPSFTM and two-stage method due to an association between treatment received, counterfactual censoring time, and prognosis. For ease of exposition, we assume the experimental treatment is beneficial, though similar arguments apply if it is harmful. The counterfactual survival model then involves shrinking survival times for all patients who receive the experimental treatment. For some patients, the event time (usually death) may not be observed – instead it is censored. For these patients, the RPSFTM and two-stage methods involve shrunken censoring times. The amount by which survival or censoring times are shrunk depends upon the size of the treatment effect and the duration for which the experimental treatment is received. Counterfactual censoring times will be prone to informative censoring bias if either/both of the two following criteria are met:

- If treatment switching decisions are related to prognostic factors;
- If the duration of treatment is related to prognostic factors.

Whilst both the RPSFTM and two-stage estimation seek to account for prognostic differences between switchers and non-switchers in their estimation of ψ, the potential for informative censoring in the counterfactual dataset remains because censoring times will be related to switching times, which may be related to prognostic factors. It has been suggested that possible bias associated with informative censoring can be avoided by breaking the dependence between the counterfactual censoring time and treatment received by re-censoring the counterfactual survival time associated with a given value of ψ (that is, $U_{i}(\psi )$ ) for all patients at the minimum of the administrative censoring time *C_i_* and $C_{i}\text{exp}\psi$ , representing the earliest possible censoring time over all possible treatment trajectories, $D_{i}^{*}(\psi )$ . $U_{i}(\psi )$ is then replaced by $D_{i}^{*}(\psi )$ if $D_{i}^{*}(\psi )<U_{i}(\psi )$ .^7,8,17^ In the context considered in this paper, where switching is from the control group onto the experimental treatment, survival or censoring times in the control group are re-censored at $D_{i}^{*}(\psi )$ (i.e. $C_{i}\text{exp}\psi$ , when the treatment prolongs survival) if this is less than the observed survival or censoring time for non-switchers (since, for patients who did not switch, $U_{i}(\psi )=T_{i}$ ), or less than counterfactual survival or censoring times $U_{i}(\psi )$ for switchers.

**Figure 1.**
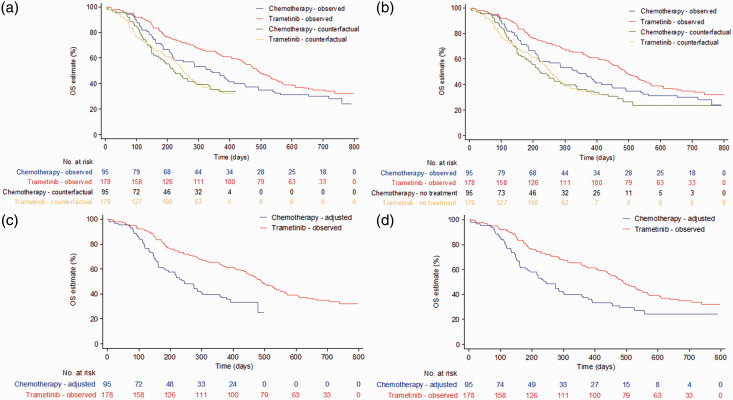
Trametinib compared to chemotherapy for metastatic melanoma: overall survival in primary efficacy population. (a) Rank-preserving structural failure time models (RPSFTM) with re-censoring; (b) RPSFTM without re-censoring; (c) two-stage method with re-censoring; (d) two-stage method without re-censoring. Adapted from Latimer et al.^11^

It is straightforward to appreciate that the greater the treatment effect ψ, the greater the impact of re-censoring, and the more control group events will be lost as the survival data are artificially censored at a time-point earlier than the follow-up times observed in the trial. A treatment effect calculated by comparing counterfactual control group survival times and observed experimental group survival times is therefore based upon shorter-term data for the control group (see Figure 1, which presents counterfactual survival curves with and without re-censoring from the trametinib example mentioned previously). If the treatment effect is not constant over time, using the re-censored survival data would result in bias if the objective is to estimate the overall longer-term treatment effect.^17^ Similarly, if short-term control group data were extrapolated to estimate long-term survival, re-censoring would be problematic if long-term trends in the hazard were not established within the timeframe of the re-censored dataset. Hence, whilst the re-censoring method aims to reduce bias associated with informative censoring in the counterfactual dataset, it may introduce another bias due to lost information, depending upon the objective of the analysis. It is common for regulatory and health technology assessment (HTA) agencies to attempt to estimate longer-term treatment effects for interventions that affect survival, with HTA agencies typically requiring estimates of lifetime treatment effects.^13–16^ There has recently been considerable interest in moving away from the hazard ratio as a summary of the treatment effect, partly because treatment effects are often observed to change over time,^25,26^ and it has been recognised that cancer populations may be characterised by complex hazard functions with turning points or important changes in trends in the longer term.^27–29^ Therefore, there is a legitimate question as to whether re-censoring or *not* re-censoring is likely to produce less bias in an adjustment analysis, given an objective of estimating long-term survival times and treatment effects.

We aim to investigate whether re-censoring or not re-censoring is likely to produce least bias in a range of realistic scenarios.

### 2.2 Simulation study design

We simulated independent datasets in which treatment switching was permitted, and in which the true survival times for each treatment option were known. Datasets with a sample size of 500 were simulated, with 2:1 randomisation in favour of the experimental group, as observed in the trametinib trial (see Figure 1). We then applied each of the switching adjustment methods with and without re-censoring, and compared the bias in their estimation of restricted mean survival time (RMST) in the control group. We focussed on control group RMST because we simulated scenarios where switching was only in the control group and therefore the objective of the adjustment analysis was to estimate counterfactual survival times for the control group. In this context, the adjustment methods do not adjust survival times in the experimental group – adjustments are made solely to control group survival times – and therefore a direct comparison of control group RMSTs, rather than a comparative treatment effect, represents the most relevant and appropriate performance measure. For each method we also calculated the empirical standard error, root mean squared error and coverage associated with estimates of control group RMST. The study was designed such that the data simulated reflected data typically observed in clinical trials in the advanced/metastatic cancer disease area. The simulation study was conducted using Stata software, version 13.1.^30^

### 2.3 Underlying survival times

A joint survival and longitudinal model was used to simultaneously generate a continuous time-dependent covariate (referred to as ‘biomarker’) and survival times,^31^ similar to the approach taken in a previous simulation study.^21^ The underlying biomarker level influenced survival and was influenced by treatment received, and observed values of the biomarker (which were subject to an error term) influenced the probability of treatment switching. Within the data-generating joint model, the longitudinal model for the underlying biomarker value for the *i*th patient at time *t* was

(2) $${\mathrm{biomarker}}_{i}(t)=\beta_{0_{i}}+\beta_{1}t+\beta_{2}t\times {\mathrm{trt}}_{i}+\beta_{3}{\mathrm{badprog}}_{i}$$

where

$$\beta_{0_{i}}∼N(\beta_{0},\sigma_{0}^{2})$$

Here $\beta_{0_{i}}$ is the random intercept, $\beta_{1}$ is the average rate of change of the biomarker for a patient in the control group, and $\beta_{1}+\beta_{2}$ is the average rate of change of the biomarker for a patient in the experimental treatment group. *trt_i_* is a binary covariate that equals 1 when the patient is in the experimental group and 0 otherwise, *badprog_i_* is a binary covariate that equals 1 when a patient has poor prognosis at baseline and 0 otherwise, and $\beta_{3}$ is the change in the intercept for a patient with a poor prognosis compared to a patient with a good prognosis. We simulated data in which biomarker observations were made at randomisation and at 21-day intervals after randomisation. Biomarker observations were subject to an error term with a standard normal distribution with mean 0 and variance $\sigma^{2}$ .

For the majority of our scenarios, we used a two-component mixture Weibull baseline survival function and the general survival simulation framework described by Crowther and Lambert.^31^ to simulate survival dependent on a time-varying biomarker. Simulating using a mixture model allows us to simulate complex hazard functions, which is important given the recognition that real-world survival data frequently do not follow standard parametric distributions.^32^ The model can be written as

(3) $$S_{0}(t)=p\text{exp}(-\lambda_{1}t^{\gamma_{1}})+(1-p)\text{exp}(-\lambda_{2}t^{\gamma_{2}})$$

where $\lambda_{1},\lambda_{2}>0$ and $\gamma_{1},\gamma_{2}>0$ are scale and shape parameters, respectively. The mixture parameter, *p*, with 0≤p≤1 , represents the contribution of the first Weibull to the overall survival model, and 1-p represents the contribution of the second Weibull. The related baseline hazard function is

(4) $$h_{0}(t)=\frac{\lambda_{1}\gamma_{1}{\mathrm{pt}}^{\gamma_{1}-1}\text{exp}(-\lambda_{1}t^{\gamma_{1}})+\lambda_{2}\gamma_{2}(1-p)t^{\gamma_{2}-1}\text{exp}(-\lambda_{2}t^{\gamma_{2}})}{p\text{exp}(-\lambda_{1}t^{\gamma_{1}})+(1-p)\text{exp}(-\lambda_{2}t^{\gamma_{2}})}$$

The linear predictor of the survival model is incorporated as follows

(5) $$h_{i}(t)=h_{0}(t)\text{exp}[\delta_{1}{\mathrm{trt}}_{i}+\eta t\times {\mathrm{trt}}_{i}+\delta_{2}{\mathrm{badprog}}_{i}+\alpha {\mathrm{biomarker}}_{i}(t)]$$

where $\delta_{1}$ is the direct effect of treatment at time 0, *η* is the rate at which the direct effect of treatment changes with time, $\delta_{2}$ is the impact of poor prognosis, and *α* is the coefficient of the underlying biomarker level.

Disease progression times were simulated to equal survival times multiplied by a value from a beta distribution with shape parameters (5, 10). We assumed that patients had consultations with their clinician every 21 days, and that disease progression was observed to have occurred at the first consultation following the actual progression event.

We simulated random entry into the study. The maximum administrative censoring time was set at 548 days (1.5 years), and patients in the control group had a random uniform entry time from 0 to 183 days – hence their administrative censoring times ranged from 365 to 548 days.

In one set of scenarios we excluded the ‘*t*’ terms from the data generating mechanism and used a single Weibull model rather than a mixture model, in order to test the different methods in instances with a simplified survivor function and a constant treatment effect (i.e. with proportional hazards) over time. Specifically, α and η were set to zero. We continued to simulate the time-dependent biomarker covariate, but this no longer affected survival. In these scenarios, re-censoring should be less prone to bias, because long-term trends in the hazard are established in the short-term and the treatment effect is constant. Comparing results from scenarios with a complex survivor function to scenarios with a simplified survivor function and treatment effect should show how sensitive methods that apply re-censoring are to these complexities.

Scenarios were ordered such that low numbers were associated with parameter values that were unlikely to result in major biases for the adjustment methods, and high numbers assessed scenarios where bias was more likely to be a problem. For instance, Scenario 1 had a simple, single Weibull survival model, a low treatment effect and a low switching proportion. Scenario 13 provides a more representative illustration of the scenarios tested, characterised by a mixture Weibull survival model and a high, time-dependent treatment effect. Parameter values for the mixture Weibull survival model and the longitudinal biomarker model in Scenario 13 were

$$\begin{array}{c} \beta_{0}=20,\sigma_{0}^{2}=1,\beta_{1}=0.04,\beta_{2}=-0.02,\beta_{3}=2.5,\\ \sigma^{2}=1,\delta_{1}=-1.30,\delta_{2}=0.3,\alpha =0.01,\lambda_{1}=0.00001,\\ \gamma_{1}=2.0,\lambda_{2}=0.00001,\gamma_{2}=0.8,p=0.5,\eta =0.003 \end{array}$$

**Figure 2.**
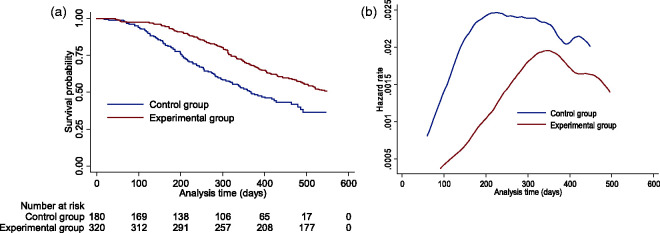
One simulated dataset from Scenario 13 with no switching: (a) Overall survival Kaplan–Meier; (b) smoothed hazard rate.

An example of the Kaplan–Meier curves and hazard function produced by the simulation model (in the absence of treatment switching) from a single simulated data set using Scenario 13 parameter values is presented in Figure 2. We simulated a hazard function that was initially low, then steadily increased before decreasing towards the end of the trial follow-up. This is similar to the data simulated in our previous study,^21^ and we believe that this is typical of the types of hazards observed in a metastatic oncology RCT setting: initial hazards are likely to be low, because trial inclusion criteria dictate that trial participants usually have relatively good prognosis. The seriousness of the disease dictates that hazards are likely to rise, before falling in the longer term as those who remain alive are of relatively better prognosis. The resulting Kaplan–Meier curves are also reminiscent of those observed in the trametinib in metastatic melanoma example presented in Figure 1.

### 2.4 Treatment effect in the experimental group

For the majority of scenarios investigated, we cannot summarise the treatment effect experienced in the experimental group using a single value, because our hazard function includes ‘*t*’ terms. Parameter values for the survival and biomarker models were chosen to generate realistic survival times and treatment effects, and these were varied in sets of scenarios to investigate the impact of different treatment effect sizes on the performance of the adjustment methods. Primarily, we investigated a treatment effect that initially increases during the period of greatest hazard, before falling in the longer term. We believe that this is representative of a realistic treatment effect, which falls in the longer term when the initial treatment effect may have worn off, or when only better prognosis patients remain alive.

In the set of scenarios that excluded the ‘*t*’ terms from the data generating mechanism, the true treatment effect was known, with $\delta_{1}$ representing the log hazard ratio. In scenarios that incorporated a time-dependent treatment effect, to give an idea of the size of the treatment effect, we calculated the ‘average’ HR and AF (andψ) by generating scenario-specific survival data for a large number of patients (1,000,000) without applying switching, and by fitting Cox and RPSFTM models to this.

### 2.5 The switching mechanism

Only patients in the control group could switch treatments, and switching could only occur during the three consultations immediately following disease progression – switching was not permitted before disease progression, to reflect the treatment switching typically seen in metastatic cancer trials.^1^ During this ‘at-risk’ period, the probability of switching declined for each individual patient with each simulated consultation, which was assumed to occur every 21 days. The probability of switching during the ‘at-risk’ period was calculated using a logistic function and depended upon the time of observed disease progression, and the observed biomarker value at that time-point. In reality, switching is highly likely to be related to prognosis and therefore in half of our simulated scenarios patients with relatively good prognosis were more likely to switch, and in the other half switching was more likely in patients with relatively poor prognosis. Switching probabilities were varied to test different switching proportions. Further details on the probability of switching in different simulated groups are presented in Appendix A, Supplementary material.

### 2.6 Treatment effect in switchers

For patients who were simulated to switch from the control treatment onto the experimental treatment, the period after switching was multiplied by a factor (*ω*) to estimate survival times incorporating the impact of switching ( $T_{z_{i}}$ ), using the following approach

(6) $$T_{z_{i}}=T_{A_{i}}+\omega \times T_{B_{i}}$$

where $T_{A_{i}}$ represents the time of switching and $T_{B_{i}}$ represents the survival time after the switch point that was simulated to occur in the absence of switching. This is the same as the accelerated failure time model presented in equation (1), but here we denote the treatment effect as ω rather than $e^{-\psi }$ .

The magnitude of *ω* was varied across scenarios to represent relative reductions in the average treatment effect in switchers compared to the experimental group (in terms of an AF) of 0% and 20%. This allowed us to test scenarios in which the ‘common treatment effect’ assumption did and did not hold. For instance, in scenarios where the common treatment effect assumption was simulated to hold, *ω* was set to equal the previously estimated scenario-specific average experimental group AF (that is, $e^{-\psi }$ ), ensuring that the treatment effect received by switchers was the same as the average effect received in the experimental group. In scenarios where a 20% treatment effect reduction in switchers was simulated, *ω* was set to equal $((e^{-\psi }-1)\times 0.8)+1$ . In scenarios where there was a time-dependent treatment effect, the common treatment effect assumption did not hold in the truest sense even when the treatment effect received by switchers was the same as the average treatment effect in the experimental group, because the treatment effect in the experimental group was time-dependent. However, in the set of scenarios that did not incorporate a time-dependent treatment effect, the common treatment effect assumption did truly hold.

### 2.7 Scenarios investigated

The simulated data generating mechanism had several variables for which values had to be assumed. These are listed in Appendix B, Supplementary material, together with details on how they were varied across scenarios. Scenarios were devised in order to cover key variables that were likely to change in trials in the real world, and also to test the sensitivities of the different adjustment methods with respect to their key assumptions. Scenarios were run varying the following characteristics:

- Switcher prognosis: good prognosis more likely to switch; poor prognosis more likely to switch;
- Common treatment effect (CTE): no CTE (20% relative treatment effect reduction received by switchers as a proportion of the average AF in the experimental group); CTE (0% relative treatment effect reduction);
- Treatment effect: low (average HR/AF/ψ under the incorrect assumption of proportional treatment effects approximately 0.80/1.13/−0.12); high (average HR/AF/ψ approximately 0.56/1.85/−0.62);
- Complexity of the survivor function and time dependency of treatment effect: simple (single Weibull model, α=0.00 , η=0.000 ); moderate (mixture Weibull model, α=0.01 , η=0.003 ); high (mixture Weibull model, α=0.01 , η=0.006 );
- Severity of disease: low (restricted mean survival in control group approximately 357 days, administrative censoring proportion approximately 40–50%); high (restricted mean survival in control group approximately 228 days, administrative censoring proportion approximately 17–25%);
- Switch proportion: low (approximately 25% of control group patients who experienced disease progression); moderate (approximately 55% of control group patients who experienced disease progression).

Using a 2 × 2 × 2 × 3 × 2 × 2 factorial design resulted in a total of 96 scenarios. The scenarios were numbered 1–96 with all levels of one factor nested inside one level of the next factor, following the order listed above. One thousand simulations were run for each scenario, with each simulation producing a dataset of 500 patients randomised at a 2:1 ratio to the experimental and control groups, respectively. Scenario settings are detailed in Appendix C, Supplementary material.

### 2.8 Adjustment methods compared

To provide context on the performance of the adjustment methods, we present results from a ‘No Switching’ analysis, representing the results of a standard ITT analysis (that is, an unadjusted estimate of control group RMST) undertaken on the simulated dataset before switching was applied. This does not represent a feasible estimator, but provides a useful upper bound for adjustment method performance which may be considered a ‘gold standard’. We also present a standard ITT analysis after switching was applied.

For the RPSFTM we used a log-rank test within the g-estimation procedure using the Stata command strbee.^33^ We included the RPSFTM with and without re-censoring (referred to as RPSFTM and RPSFTMnr, respectively).

We applied the two-stage method using a Weibull model, used disease progression as the secondary baseline time-point, and included covariates for switching, baseline prognosis group, observed biomarker value at time 0, observed time-to-disease progression, and observed biomarker value at disease progression. We included the two-stage method with and without re-censoring (referred to as TSE and TSEnr respectively).

### 2.9 Performance measures

We used control group restricted mean survival time (RMST) as our true value, or estimand, upon which to base our performance measures. Our simulated survival function was not analytically tractable, so for each scenario we simulated data for 1,000,000 patients without incorporating treatment switching, and we estimated the RMST at 548 days (the maximum administrative censoring time in the simulated datasets). Because this value is the product of a simulation rather than a calculation, it is prone to error, but this is likely to be extremely minimal given the large number of patients simulated.

To estimate RMST at 548 days for each of the adjustment methods, we could not simply calculate the area under the counterfactual Kaplan–Meier curve because this may restrict the mean estimation to too short a time period, particularly for methods that apply re-censoring. Instead, we used what we believe is the most appropriate approach given the context that these methods are usually used in – that is, for health technology assessment. TSE, TSEnr, RPSFTM and RPSFTMnr each provide counterfactual datasets, to which we fitted flexible parametric models in order to obtain the survivor function extrapolated to 548 days. The Stata command stpm2 was used to fit the models on the log cumulative hazard scale, with three knots placed at equally spaced centiles of the distribution of the log survival times.^34^ Where the final observed survival time was less than 548 days, the RMST at 548 days was estimated through a linear extrapolation from the last knot. This is in line with recommendations made in the UK for undertaking survival modelling in the absence of proportional hazards.^35,36^ To estimate confidence intervals (CIs), counterfactual datasets were derived for the lower and upper 95% CIs of the estimated treatment effect (ψ ) for each of the adjustment methods. Then flexible parametric models were fitted as described above to estimate 95% CIs for RMST at 548 days.

We evaluated the performance of methods according to the percentage bias in their estimate of control group RMST at 548 days. Percentage bias was estimated by taking the difference between the mean estimated RMST and the true RMST and expressing this as a percentage of true RMST.^37^ The root mean squared error (RMSE) of the percentage bias was calculated to provide information on the variability of estimates in combination with percentage bias. The empirical standard error (SE) of the RMST estimate was also calculated for each method, as was coverage, defined as the proportion of simulations where the 95% confidence interval of the RMST contained the true RMST. Convergence was measured, defined as the proportion of times that each method resulted in an estimate of control group RMST. Percentage bias, RMSE, empirical SE and coverage were calculated based upon simulations in which convergence occurred. Monte Carlo (MC) standard errors were also calculated for each performance measure, for each method.

## 3 Results

First we present detailed results from four important scenarios, chosen to highlight key drivers of the results. We then summarise how these results relate to the full range of scenarios tested. Finally, we draw together key findings on within-method performance – comparing TSE to TSEnr and RPSFTM to RPSFTMnr, followed by key findings on between-method performance.

A summary table describing the characteristics of each scenario is presented in Appendix D, Supplementary material. Supplementary material Appendices E, F and G present the percentage bias, empirical standard error, and RMSE, respectively, across all scenarios for each method.

### 3.1 Detailed results from key scenarios

Table 1 presents detailed results from Scenarios 9 and 13. Scenario 9 incorporated a low switch proportion, a low treatment effect, low disease severity, a moderately complex survivor function, and violated the common treatment effect assumption. As expected, the ITT analysis estimated a higher control group RMST than would have been observed in the absence of treatment switching, but bias was low – equivalent to a percentage bias of 1.2%. The adjustment methods – except the RPSFTM – led to reduced bias. The RPSFTM and TSE analyses that applied re-censoring both underestimated control group RMST, with the level of bias more appreciable for the RPSFTM (percentage bias −1.2%, compared to −0.3% for TSE). In contrast, RPSFTMnr and TSEnr analyses overestimated control group RMST (percentage bias 0.4% for the RPSFTMnr and 0.7% for the TSEnr).

**Table 1.** Scenarios 9 and 13 – performance measures for estimation of control arm RMST.

Scenario details	Method	Percentage bias	Empirical SE of percentage bias	RMSE of percentage bias	Coverage (%)	Convergence (%)
Scenario number: 9 True RMST: Control: 357 Experimental: 391 Mean switch: 25% True ave. HR: 0.81 True ave. AF: 1.19 Mean censored: 40% Switcher treatment effect: 20% reduction	No switching	0.8	3.6	3.6	95.6	100
	ITT	1.2	3.6	3.8	94.8	100
	TSE	−0.3	4.3	4.3	49.4	100
	TSEnr	0.7	3.7	3.7	29.2	100
	RPSFTM	−1.2	5.5	5.6	37.7	100
	RPSFTMnr	0.4	4.0	4.1	19.4	100
	min/max MC error	0.1/0.2	0.1/0.1	0.1/0.2	0.6/1.6	–
Scenario number: 13 True RMST: Control: 357 Experimental: 430 Mean switch: 25% True ave. HR: 0.57 True ave. AF: 1.53 Mean censored: 48% Switcher treatment effect: 20% reduction	No switching	0.1	3.7	3.7	94.6	100
	ITT	2.8	3.6	4.5	90.0	100
	TSE	−1.6	5.7	5.9	53.5	100
	TSEnr	1.4	3.7	4.0	24.7	100
	RPSFTM	−3.8	7.2	8.2	36.3	100
	RPSFTMnr	0.9	4.1	4.2	17.2	100
	min/max MC error	0.1/0.2	0.1/0.2	0.1/0.2	0.7/1.6	–

RMST: restricted mean survival time; HR: hazard ratio; AF: acceleration factor; SE: standard error; RMSE: root mean squared error; MC: Monte-Carlo; ITT: intention to treat; TSE: two-stage estimation; TSEnr: two-stage estimation without re-censoring; RPSFTM: rank preserving structural failure time model; RPSFTMnr: rank preserving structural failure time model without re-censoring.

The only substantive difference between Scenario 9 and Scenario 13 was that the treatment effect was higher in Scenario 13, with an average HR of 0.57, compared to 0.81 in Scenario 9. Percentage bias increased appreciably for the ITT analysis and for each of the adjustment methods in Scenario 13. Methods that applied re-censoring continued to underestimate control group RMST (percentage bias −1.6% and −3.8% for TSE and RPSFTM, respectively), and methods that did not apply re-censoring continued to overestimate control group RMST (1.4% and 0.9% for TSEnr and RPSFTMnr, respectively).

Table 2 presents detailed results from Scenarios 57 and 61. These were similar to Scenarios 9 and 13, with the only substantive difference the switching proportion, which was increased to approximately 57% of at-risk patients (39% of all control group patients).

**Table 2.** Scenarios 57 and 61 – performance measures for estimation of control arm RMST.

Scenario details	Method	Bias (percentage of true RMST)	Empirical SE (percentage of true RMST)	RMSE (percentage of true RMST)	Coverage (%)	Convergence (%)
Scenario number: 57 True RMST: Control: 357 Experimental: 391 Mean switch: 57% True ave. HR: 0.81 True ave. AF: 1.19 Mean censored: 40% Switcher treatment effect: 20% reduction	No switching	0.2	3.6	3.6	96.0	100
	ITT	2.6	3.6	4.4	89.5	100
	TSE	−1.1	5.2	5.3	67.2	100
	TSEnr	0.8	4.0	4.1	48.6	100
	RPSFTM	−2.0	7.3	7.6	66.5	100
	RPSFTMnr	0.7	5.0	5.0	46.7	100
	min/max MC error	0.1/0.2	0.1/0.2	0.1/0.2	0.6/1.6	–
Scenario number: 61 True RMST: Control: 357 Experimental: 430 Mean switch: 57% True ave. HR: 0.57 True ave. AF: 1.53 Mean censored: 49% Switcher treatment effect: 20% reduction	No switching	0.2	3.8	3.8	94.3	100
	ITT	6.5	3.6	7.5	58.8	100
	TSE	−1.9	6.6	6.9	66.8	100
	TSEnr	3.0	4.0	5.0	33.6	100
	RPSFTM	−5.3	9.3	10.7	56.8	100
	RPSFTMnr	2.1	5.1	5.5	36.0	100
	min/max MC error	0.1/0.3	0.1/0.2	0.1/0.3	0.7/1.6	–

RMST: restricted mean survival time; HR: hazard ratio; AF: acceleration factor; SE: standard error; RMSE: root mean squared error; MC: Monte-Carlo; ITT: intention to treat; TSE: two-stage estimation; TSEnr: two-stage estimation without re-censoring; RPSFTM: rank preserving structural failure time model; RPSFTMnr: rank preserving structural failure time model without re-censoring

The increased switching proportion had an important impact, leading to increased bias, with the relative effect on the different adjustment methods dependent on the size of treatment effect. Comparing Scenario 57 to Scenario 9 (scenarios in which the treatment effect was small), the impact of the increased switching proportion was most important for the adjustment methods that re-censored, with percentage bias approximately doubled in Scenario 57 (percentage bias −1.1% and −2.0% for TSE and RPSFTM, respectively). Percentage bias also increased for TSEnr and RPSFTMnr in Scenario 57, but the difference was not as substantial (percentage bias 0.8% and 0.7% for TSEnr and RPSFTMnr respectively). Bias was further increased for each of the adjustment methods in Scenario 61, in which both the treatment effect and the switching proportion were high, but the impact in this scenario was relatively more important for the adjustment methods that did not re-censor. For TSEnr and RPSFTMnr bias approximately doubled compared to Scenario 13 (percentage bias 3.0% and 2.1% for TSEnr and RPSFTMnr, respectively), whereas for TSE and RPSFTM the increase in bias was more marginal (percentage bias −1.9% and −5.3% for TSE and RPSFTM, respectively).

In Scenarios 9, 13, 57 and 61, the TSE, TSEnr and RPSFTMnr methods produced similarly low levels of bias, with the RPSFTM producing a higher level of bias. The direction of the biases remained the same in each scenario – applications that re-censored resulted in negative bias and those that did not re-censor resulted in positive bias. TSE, TSEnr and RPSFTMnr produced lower percentage bias than the ITT analysis, but the RPSFTM did not when the switching proportion was low. Levels of variability associated with the different adjustment methods differed importantly. Higher levels of bias were not always associated with higher RMSEs. For instance, in each scenario TSEnr produced least RMSE aside from the gold standard ‘no switching’ analysis, and both the TSEnr and RPSFTMnr produced appreciably lower RMSE than TSE and RPSFTM, even when the methods that re-censored resulted in lower percentage bias. This reflects the fact that the empirical SE of the percentage bias differed substantially between methods. Two-stage and RPSFTM methods that re-censored produced empirical SEs that were approximately 50% larger than those associated with methods that did not re-censor.

Coverage was poor for all the adjustment methods, although methods that re-censored provided better coverage than those that did not. Convergence was achieved with all of the adjustment methods in each of the scenarios.

### 3.2 Results from other scenarios

**Figure 3.**
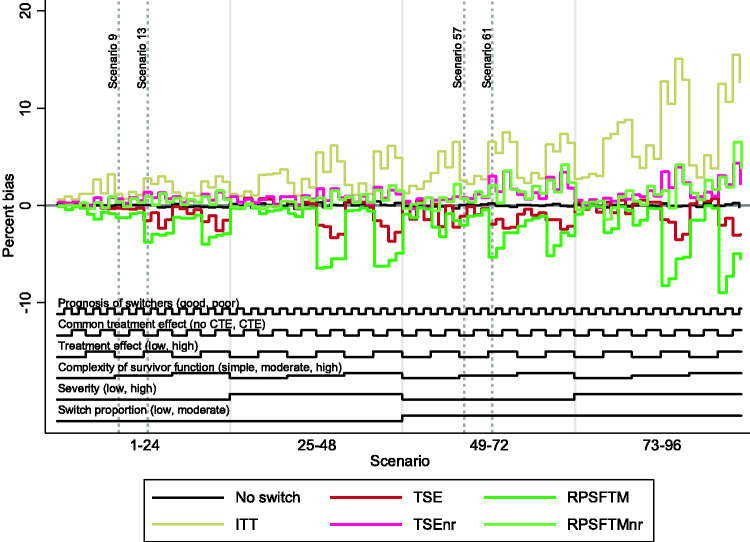
Percentage bias across all scenarios. ITT: intention to treat; TSE: two-stage estimation; TSEnr: two-stage estimation without re-censoring; RPSFTM: rank preserving structural failure time model; RPSFTMnr: rank preserving structural failure time model without re-censoring; CTE: common treatment effect.

**Figure 4.**
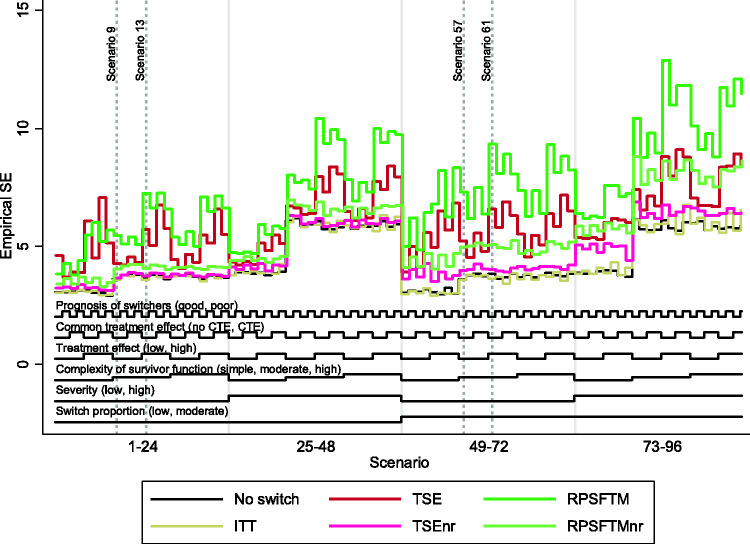
Empirical standard error across all scenarios. ITT: intention to treat; TSE: two-stage estimation; TSEnr: two-stage estimation without re-censoring; RPSFTM: rank preserving structural failure time model; RPSFTMnr: rank preserving structural failure time model without re-censoring; CTE: common treatment effect; SE: standard error.

**Figure 5.**
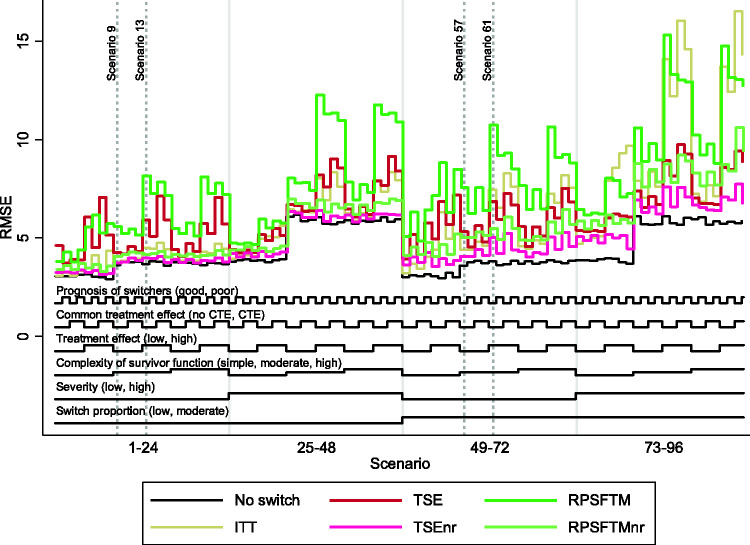
Root mean squared error across all scenarios. ITT: intention to treat; TSE: two-stage estimation; TSEnr: two-stage estimation without re-censoring; RPSFTM: rank preserving structural failure time model; RPSFTMnr: rank preserving structural failure time model without re-censoring; CTE: common treatment effect; RMSE: root mean squared error.

The overall patterns in our results are illustrated in Figures 3 to 5, which present nested loop plots for percentage bias, empirical SE and RMSE.^38^ More detailed barplots for each of these performance measures are presented in Supplementary material Appendices E, F and G. The results presented above provide a good basis for reporting the results of the remaining scenarios – particularly those observed in scenarios where the complexity of the survivor function was moderate or high. The characteristics that had the most impact on the performance of the adjustment methods were the complexity of the survivor function, the switching proportion, and the size of the treatment effect.

When the survivor function was simple, with a constant treatment effect over time, the adjustment methods generally produced similar and low levels of bias, with the only exception being the RPSFTM which produced higher levels of bias when there was a high, non-common treatment effect and a high switching proportion. The range of empirical SE and RMSE produced by the different adjustment methods was narrower than in other scenarios, but TSEnr consistently produced the lowest values. RPSFTM produced negative bias in the vast majority of scenarios and TSEnr usually produced positive bias. The direction of bias was more varied for the TSE and RPSFTMnr methods. TSE generally produced negative bias when poor prognosis patients were more likely to switch, and generally produced positive bias when good prognosis patients were more likely to switch. RPSFTMnr produced positive bias in the majority of these scenarios, but often produced negative bias when the common treatment effect assumption was violated.

When the complexity of the survivor function was moderate or high, with a decreasing treatment effect over time, the performance of the adjustment methods varied much more widely, with bias approximately doubled in size compared to that observed in scenarios with a simple survivor function and a constant treatment effect. TSEnr produced positive bias (overestimating control group RMST) in all 64 of these scenarios. RPSFTMnr produced positive bias in all but four scenarios – and where it produced negative bias this was of a similar size to the associated MC error. RPSFTM produced negative bias (underestimating control group mean survival) in all of 64 scenarios, and TSE produced negative bias in the vast majority of scenarios, but occasionally produced positive bias when the treatment effect was low (though in these scenarios the positive bias was often of a similar size to the associated MC error).

Percentage bias was increased for all methods when the treatment effect was high. Similarly, percentage bias was increased for all methods when the switching proportion was moderate as opposed to low, although the impact was much more substantial for methods that did not apply re-censoring. When the switching proportion was low, the size of the treatment effect was less important for TSEnr and RPSFTMnr, but remained a crucial determinant of the bias associated with TSE and RPSFTM.

Based upon Figure 3, high disease severity appears to be an important driver of bias. However, it is important to note that whilst the average hazard ratio associated with treatment was (by design) held the same in equivalent scenarios characterised by high and low disease severity, the average acceleration factor was higher in high severity scenarios. Given that the RPSFTM and TSE methods use accelerated failure time models, this is important and means that the impact of disease severity cannot be interpreted in isolation – the observed increase in bias may be due to the increased disease severity, or may be due to the increased acceleration factor.

The common treatment effect characteristic had an impact on the RPSFTM (bias reduced when there was a common treatment effect) and RPSFTMnr (bias increased when there was a common treatment effect), but it did not affect the TSE and TSEnr methods. Whether poor prognosis patients or good prognosis patients were more likely to switch did not have an impact on RPSFTM and TSE methods. The impact on TSEnr and RPSFTMnr was larger – both produced reduced bias when poor prognosis patients were more likely to switch. In general, the absolute impact of switching was reduced in scenarios where poor prognosis patients were more likely to switch.

### 3.3 Key findings – within method comparisons

As shown by Figure 3, and as previously described, in scenarios where the complexity of the survivor function was moderate or high, with reducing hazards and a reducing treatment effect in the longer-term, the level of bias associated with TSE and TSEnr methods was often similar but, importantly, was in opposite directions. TSE consistently underestimated control group RMST, and TSEnr consistently overestimated control group RMST. In these scenarios only the size of the treatment effect and the switch proportion had substantial impacts on the bias associated with these methods. The TSEnr performed better when the switching proportion was low and, independently of this, when the treatment effect was small. The TSE method was relatively unaffected by the switch proportion but its performance was markedly improved when the treatment effect was low. The method that produced least bias varied, but, in general, TSEnr was more likely to produce least bias in scenarios where the treatment effect was high and the switching proportion was low – in other scenarios TSE was more likely to produce least bias. However, TSEnr produced lower empirical SE and RMSE across all scenarios.

In scenarios in which the survivor function was simplified, with a constant treatment effect, both methods produced substantially lower bias – with TSE generally producing marginally lower levels of bias (but higher RMSE) than TSEnr.

The key findings associated with the RPSFTM and RPSFTMnr methods were similar – but not identical – to those found for TSE and TSEnr. In scenarios in which the complexity of the survivor function was moderate or high, biases were again in opposing directions, with the RPSFTM consistently underestimating, and RPSFTMnr consistently overestimating control group RMST. The size of the treatment effect and the switching proportion were again the most important determinants of bias, with the size of the treatment effect being most important for RPSFTM and the switching proportion being most important for RPSFTMnr. However, the method that produced least bias was much less variable, with the RPSFTMnr producing lower bias than RPSFTM in the vast majority of scenarios, with this only changing in some scenarios in which there was a low and common treatment effect. The RPSFTMnr produced lower RMSE and empirical SE in all scenarios.

In scenarios in which the survivor function was simplified, bias was again substantially reduced for both methods, but RPSFTMnr continued to generally produce lower bias than RPSFTM.

### 3.4 Key findings – between method comparisons

Across all scenarios the TSE, TSEnr and RPSFTMnr methods often produced bias of similar size, though TSEnr and RPSFTMnr consistently overestimated control group RMST (thus underestimating treatment effects) and TSE consistently underestimated control group RMST (thus overestimating treatment effects). The RPSFTM consistently produced higher levels of bias unless there was a low and common treatment effect. The TSE produced least percentage bias most often, particularly in scenarios characterised by a moderate or highly complex survivor function and a moderate switching proportion, or a low switching proportion combined with a low treatment effect (see Table 3). The RPSFTMnr produced least bias in the majority of scenarios when the switching proportion was low combined with a high treatment effect. However, TSEnr produced the lowest RMSE across all scenarios and methods that did not apply re-censoring always produced lower empirical SE and RMSE than those that did apply re-censoring.

**Table 3.** Methods producing least bias.

	Complexity of survivor function: simple				Complexity of survivor function: moderate/high				Total
	Low switch proportion		Moderate switch proportion		Low switch proportion		Moderate switch proportion		
	Low treatment effect	High treatment effect	Low treatment effect	High treatment effect	Low treatment effect	High treatment effect	Low treatment effect	High treatment effect	
Scenarios	1–4, 25–28	5–9, 29–32	49–52, 73–76	53–56, 77–80	9–12, 17–20, 33–36, 41–44	13–16, 21–24, 37–40, 45–48	57–60, 65–68, 81–84, 89–92	61–64, 69–72, 85–88, 93–96	All
ITT	0	0	0	0	0	0	0	0	0
TSE	2	4	2	3	8	0	9	9	36
TSEnr	0	1	2	2	2	3	1	2	13
RPSFTM	1	1	3	2	2	0	3	0	12
RPSFTMnr	5	3	1	1	4	13	3	5	35

ITT: intention to treat; TSE: two-stage estimation; TSEnr: two-stage estimation without re-censoring; RPSFTM: rank preserving structural failure time model; RPSFTMnr: rank preserving structural failure time model without re-censoring.

## 4 Discussion

Our study demonstrates that adjustment analyses should be conducted both with and without re-censoring. Re-censored and non-re-censored analyses are likely to often produce bias in opposing directions, potentially providing additional information on where the true treatment effect is likely to lie. There are three main factors that dictate the size and direction of bias associated with TSE, TSEnr, RPSFTM and RPSFTMnr methods when the objective is to estimate long-term survival times: the complexity of the survivor function; the size of the treatment effect; and the switching proportion. In addition, we found that the RPSFTM produced higher levels of bias than the other adjustment methods in the majority of scenarios.

When the survivor function is simple, with monotonic hazards over time and a constant treatment effect, re-censoring is relatively unimportant. All methods generally produce low levels of bias and differences in their estimates are generally small and are driven mainly by whether or not methodological assumptions such as the common treatment effect assumption hold. Re-censoring in and of itself does not result in bias if accurate extrapolations can be achieved from shorter-term data, which is more likely when survivor functions are characterised by long-term trends that are established in the short-term. On a case-by-case basis, this may not be straightforward to determine, but could be informed by comparisons between the re-censored and the non-re-censored dataset, and by analysing the long-term hazards observed in the experimental group – as trends in the hazards observed in long-term survivors in the experimental group might also be expected in long-term survivors in the control group.

Complex hazard functions with changing trends over time and turning points have been presented in the literature for breast cancer,^27,28^ and the presence of long-term survivors has been noted for several other cancer types, such as melanoma, head and neck cancer and myeloid leukaemia, particularly with the advent of new immuno-oncology treatments.^29^ It therefore seems likely that there will often be important time-dependent changes in the hazard function representing cohorts of cancer patients. This is why we concentrated our simulations on scenarios with complex hazard functions and it is therefore important to focus discussion of the re-censoring issue on circumstances where the survivor function is complex.

When the survivor function is complex, with a hazard that initially rises before falling over time, and with a time-dependent treatment effect, methods that re-censor are likely to produce bias in opposite directions to methods that do not re-censor when estimating longer term mean survival. In isolation, this is an important finding. We have demonstrated that extrapolating from re-censored data leads to underestimates of long-term survival in groups affected by switching. Conversely, the informative censoring associated with not re-censoring leads to overestimates of long-term survival in groups affected by switching.

Further, we have shown that the most important determinant of performance for methods that re-censor is the size of the treatment effect. This is logical – re-censoring results in more lost information when the treatment effect is high, reducing the probability that long-term trends in the hazard function are established within the re-censored dataset. For methods that do not re-censor, the size of the treatment effect and the switching proportion are of most importance. This also makes sense – not re-censoring leads to less informative censoring when the switching proportion is low, and to less substantial informative censoring when the treatment effect is small.

Our intention was primarily to compare re-censored and non-re-censored analyses within the RPSFTM and TSE classes. However, our results also provide new information allowing us to update the comparison between these two classes. Whilst no single method produced least percentage bias consistently across all scenarios, TSEnr produced the lowest empirical SE and RMSE of the adjustment methods in all 96 scenarios, suggesting that when bias and variability are considered together, it consistently represented the optimal method. In addition, the RPSFTM (with re-censoring) performed substantially worse than other adjustment methods in a subset of scenarios, allowing scenarios to be identified in which this method should not be relied upon.

The RPSFTM with re-censoring consistently produced negative bias (overestimating the treatment effect) and performed substantially worse than other adjustment methods in scenarios with a high treatment effect and a complex survivor function, particularly when there was not a common treatment effect. When the survivor function is characterised by hazards that begin to fall in the longer term, re-censoring causes a negative bias if the trend in the hazards is not established before the re-censoring time-point. In addition, when switchers receive a decreased treatment effect, the RPSFTM – which assumes a common treatment effect – will over-adjust survival times for switchers, causing an additional negative bias. Hence, the RPSFTM with re-censoring is prone to two sources of negative bias in scenarios such as those investigated in this study – two-thirds of scenarios incorporated a complex hazard function with decreasing hazards in the longer term, and half incorporated a violation of the common treatment effect assumption, where switchers received a reduced treatment effect. The TSE is only prone to one of these sources of bias because it does not assume a common treatment effect, which explains why the TSE consistently produced more conservative estimates of restricted mean survival than the RPSFTM.

If, in reality, hazards are expected to decline over time, both the RPSFTM and TSE with re-censoring are likely to lead to underestimates of longer-term control group survival. If, in addition, switchers receive a decreased treatment effect, the RPSFTM with re-censoring is likely to lead to even more serious underestimates of longer-term control group survival. Overall, the TSE appears to represent a better method than the RPSFTM for adjusting for treatment switching unless the treatment effect is common and constant, provided the switching mechanism matches the requirements of the TSE method.

When re-censoring is not applied within RPSFTM and TSE adjustment methods, they are no longer exposed to the negative bias associated with a loss of longer-term information in the presence of hazards that decrease over time. The RPSFTMnr remains exposed to the negative bias associated with a non-common treatment effect and both methods become exposed to bias associated with informative censoring. Originally we hypothesised that informative censoring would be associated with positive bias (overestimates of control group survival) when poor prognosis patients were more likely to switch treatments – because more poor prognosis patients would be censored at earlier time-points than good prognosis patients. Conversely, when good prognosis patients were more likely to switch, we expected that not re-censoring would lead to negative bias (underestimates of control group survival), because good prognosis patients would generally be censored at earlier time-points. In fact, in scenarios where there was a complex survivor function, RPSFTMnr and TSEnr almost always produced positive bias, irrespective of the prognosis of switchers.

After thorough investigation, we conclude that this will occur when there are any non-switching, long-term survivors (see Appendix H, Supplementary material for more details). These patients most influence the impact of informative censoring, because re-censoring primarily affects the right-hand side of the Kaplan–Meier curve. It is this that causes the methods that do not re-censor to result in bias in the opposite direction to that produced by methods that re-censor.

Which bias is greater depends partly on scenario characteristics and partly on whether the TSE or RPSFTM adjustment method is used. In our simulations TSE and TSEnr often produced similarly low levels of bias, in opposite directions, but there was a trend towards TSE being likely to produce less bias than TSEnr unless the treatment effect was high (HR approximately 0.56 as opposed to 0.80) and the switching proportion was low (approximately 25% of eligible patients as opposed to 55%). However, the TSEnr always produced lower empirical SE and RMSE than TSE and therefore may still be preferred when the aim is to estimate long-term treatment effects.

More confident conclusions may be made about which of the RPSFTM and RPSFTMnr analyses are likely to produce least bias. When there is a complex survivor function with reducing long-term hazards and when the common treatment effect assumption does not hold, the RPSFTMnr is subject to opposing forces of bias – violation of the common treatment effect assumption induces negative bias, whereas informative censoring is likely to cause positive bias. Conversely, the RPSFTM is prone to the dual negative biases associated with re-censoring and a non-common treatment effect. Whilst the RPSFTM is likely to result in appreciable negative bias in these scenarios, the direction of bias associated with the RPSFTMnr depends upon the extent to which negative and positive biases cancel out. Given that these biases are likely to cancel out to some extent, it seems reasonable to conclude that the RPSFTMnr is likely to produce lower bias than the RPSFTM in these scenarios – as was almost exclusively the case in our simulations. The RPSFTMnr also consistently produced lower empirical SE and RMSE than RPSFTM and therefore may generally be preferred when the aim is to estimate long-term treatment effects. The competing biases associated with the RPSFTMnr also explain why this method generally produced marginally lower percentage bias than the TSEnr in scenarios where the common treatment effect assumption did not hold, and very similar levels of percentage bias when the common treatment effect assumption did hold.

We are aware of three studies that have presented analyses adjusting for treatment switching both with and without re-censoring, or which have investigated the impact of re-censoring. White et al.^17^ presented RPSFTM analyses undertaken on the Concorde trial of immediate versus deferred zidovudine for patients with HIV. The analysis without re-censoring led to more conservative estimates of the treatment effect and the authors observed that the treatment effect appeared to decrease over time. They concluded that their re-censored analysis may have overestimated the treatment effect, whilst their non-re-censored analysis may have produced an underestimate because switchers appeared to have a better prognosis than non-switchers.^17^ Latimer et al. reported an adjustment analysis applied to an RCT comparing trametinib and chemotherapy in patients with metastatic melanoma.^11^ RSPFTM and two-stage analyses which excluded re-censoring produced the most conservative estimates of the treatment effect. The authors found evidence of a decreasing treatment effect over time, and concluded that the analyses that excluded re-censoring were likely to be least biased. The pattern in these results is identical to that seen in our study. This was not the case in White and Goetghebeur’s analysis of an RCT comparing two anti-hypertensive treatments affected by treatment switching. Heavily re-censored analyses resulted in less optimistic estimates of the treatment effect, because the treatment effect only became apparent in the long term.^20^ It is possible that in some situations the treatment effect may rise and then fall over time – in fact this was the pattern simulated in our scenarios (see Figure 2). If re-censoring leads to analyses being based on data observed before the treatment effect becomes apparent, underestimates of the long-term treatment effect may result. This was not the case in our simulations, but with a more delayed treatment effect it is conceivable.

Previous authors have noted that failing to re-censor may result in a small bias but a large gain in precision.^17^ We found that failing to re-censor often led to a large gain in precision and reduced bias. RMSE and empirical SE were substantially reduced when re-censoring was excluded from RPSFTM and TSE analyses, highlighting important advantages associated with not re-censoring.

Our study has limitations. We sought to investigate many realistic scenarios, but a simulation study can never be exhaustive. Our choice of endpoint could also be questioned – whilst we used restricted means to limit the impact of extrapolation on our results, extrapolation may still have introduced an additional bias for methods that re-censored, because for these methods extrapolation was required to estimate RMST at 548 days. However, this bias is attributable to the adjustment method, because re-censoring enforces a loss of information which makes the estimation of long-term survival more difficult. Because we used restricted rather than unrestricted mean survival as our performance measure, the impact of extrapolation is limited in our study. HTA agencies typically require estimates of unrestricted mean survival. We expect that all adjustment methods would have produced increased bias if we had used this as our performance measure – with methods that re-censor likely to be most seriously affected owing to the associated loss of information. However, this would have shifted the emphasis of our study onto methods for extrapolating survival data, rather than methods for adjusting for treatment switching. We could instead have chosen to use a mean restricted to a shorter time-period, to prevent the results of re-censored analyses from being affected by extrapolation. However, given that our intention is to help inform the choice of adjustment method used primarily within HTA analyses, we deemed it of little value to assess the performance of the different adjustment methods in estimating short-term treatment effects. Another option would have been to compare bias in the estimated difference in mean survival in the control group and the experimental group. We felt that concentrating solely on control group mean survival provided a more direct assessment of the adjustment methods, since they only made adjustments to the control group. However, it is relevant to note that this may make biases seem less important than they actually are. For instance, in Scenarios 13 and 61, a 1% bias in control group RMST is equivalent to a bias of approximately 5% in the difference between control group and experimental group RMST. In Scenarios 9 and 57, this is further inflated – with a 1% bias in control group RMST equivalent to a bias of approximately 10% in the estimated RMST difference.

Also, we recognise that results of survival analyses are usually summarised as hazard ratios. The majority of our scenarios had non-proportional hazards so HRs were inappropriate for measuring performance. Despite this, we did calculate ‘average’ HRs to allow assessment of adjusted HRs. This is presented in Appendix I, Supplementary material. We found that estimates of HRs were prone to higher levels of bias than estimates of restricted mean survival – particularly if there is a complex survivor function and a time-dependent treatment effect and re-censoring is used. This should be borne in mind if adjustment analyses are summarised using HRs.

Finally, as with previous simulation studies on switching adjustment methods, we did not incorporate bootstrapping for confidence intervals of RMST estimates.^21,22^ Coverage levels associated with the adjustment analyses are correspondingly poor because confidence intervals only took into account uncertainty in the treatment effect – not the uncertainty in the underlying survival distribution. For this reason, we have not presented detailed coverage results. In reality, the entire adjustment process should be bootstrapped to obtain appropriate confidence intervals.

We have shown that both re-censored and non-re-censored adjustment analyses are prone to bias, depending upon scenario characteristics. Our study provides valuable information on the likely direction and extent of these biases, and on their variability. Analyses that exclude re-censoring are likely to produce underestimates of the treatment effect, irrespective of the perceived prognosis of switchers. Re-censored analyses are likely to produce overestimates of the treatment effect if the treatment effect decreases over time, especially RPSFTM analyses if switchers receive a reduced treatment effect. Whilst we found that TSE, TSEnr and RPSFTMnr analyses often produced similar levels of bias, the choice of method remains important because the bias associated with re-censored and non-re-censored analyses is likely to be in opposite directions. Our results can be used to enable better interpretation of treatment switching adjustment analyses, by helping determine a range in which the true treatment effect is likely to lie. We suggest that analyses should be conducted with and without re-censoring, and that re-censoring should not always represent the default approach when the objective is to estimate long-term survival times and treatment effects. In the context of HTA, decision-makers are likely to choose one adjustment method to inform their base case estimate of the cost-effectiveness of a novel treatment. However, being aware of the sensitivity of this estimate to the adjustment method chosen could importantly influence decision-making, providing either reassurance or concern. This additional information could therefore helpfully impact upon decisions made on access to novel treatments around the world.

## Supplemental Material

Supplemental material for Causal inference for long-term survival in randomised trials with treatment switching: Should re-censoring be applied when estimating counterfactual survival times? Supplemental material for Causal inference for long-term survival in randomised trials with treatment switching: Should re-censoring be applied when estimating counterfactual survival times? by NR Latimer, IR White, KR Abrams and U Siebert in Statistical Methods in Medical Research
