# Supplementary material for: Causal inference for long-term survival in randomised trials with treatment switching: Should re-censoring be applied when estimating counterfactual survival times?
Source: Stat Methods Med Res. 2018 Jun 25;28(8):2475–93. doi: 10.1177/0962280218780856 (PMC6676341; doi:10.1177/0962280218780856)
Supplement: Supplemental material for Causal inference for long-term survival in randomised trials with treatment switching: Should re-censoring be applied when estimating counterfactual survival times? [file Supplemental_material.pdf]

APPENDICES

Appendix A: Treatment switching probabilities

Table A1 presents the probability of switching for different patient groups at different time-points in Scenarios 1, 3, 5 and 7. Higher group numbers represent higher values for that group (that is, ‘time to progression group’ 0 are the control group patients that had time-to-progression times in the lowest 33.3% of the control group). Note however that these groups only refer to patients who became ‘at-risk’ of switching – that is, those control group patients that survived for longer than 21 days. Hence the lowest 33% represent the lowest third of the at-risk group, not the control group as a whole. Switching could happen at the three consultations immediately following disease progression, with the probability of switching declining in each consultation.

Table A1: Probability of treatment switch by prognostic groups and consultation – Good prognosis more likely to switch. Scenarios 1, 3, 5, 7

Consultation 1 (post progression)		Biomarker group at progression		
		0	1	2
Time to progression group	0	0.01	0.02	0.07
	1	0.03	0.06	0.18
	2	0.11	0.20	0.46
Consultation 2 (post progression)		Biomarker group at progression		
		0	1	2
Time to progression group	0	0.01	0.02	0.5
	1	0.02	0.05	0.15
	2	0.09	0.17	0.41
Consultation 3 (post progression)		Biomarker group at progression		
		0	1	2
Time to progression group	0	0.01	0.01	0.03
	1	0.02	0.03	0.10
	2	0.06	0.11	0.30

In Scenario 1 the mean switching proportion in the control group across the 1,000 simulations was 23.5%, which was equivalent to 24.4% of control group patients who became at-risk of switching – i.e. those that experienced disease progression.

Table A2 presents the probability of switching for different patient groups at different time-points in Scenarios 2, 4, 6 and 8. In these scenarios, poor prognosis patients were more likely to switch. In Scenario 17 the mean switching proportion in the control group across the 1,000 simulations was 26.7%, which was equivalent to

27.7% of control group patients who became at-risk of switching – i.e. those that experienced disease progression.

Table A2: Probability of treatment switch by prognostic groups and consultation – Poor prognosis more likely to switch. Scenarios 2, 4, 6 and 8

Consultation 1 (post progression)		Biomarker group at progression		
		0	1	2
Time to progression group	0	0.40	0.25	0.09
	1	0.16	0.09	0.03
	2	0.05	0.03	0.01
Consultation 2 (post progression)		Biomarker group at progression		
		0	1	2
Time to progression group	0	0.35	0.21	0.07
	1	0.13	0.07	0.02
	2	0.04	0.02	0.01
Consultation 3 (post progression)		Biomarker group at progression		
		0	1	2
Time to progression group	0	0.25	0.14	0.05
	1	0.09	0.05	0.01
	2	0.03	0.01	0.00

All probabilities in Table A1 and Table A2 were increased when investigating higher switching scenarios (i.e. in Scenarios 49-56). Probabilities were adjusted in scenarios where the complexity of the survivor function was moderate or complex in order to maintain similar average switch proportions with altered survival distributions.

Appendix B: Scenario parameter values

In Table B1, values for each variable in Scenario 1 are quoted, as are alternative values for the 16 base scenarios.

Table B1: Simulated scenarios – Parameter values and alternatives tested

Variable	Value (Scenario 1)	Alternative Values
Sample size	500 (2:1 randomisation)	-
Number of prognosis groups (prog)	2	-
Probability of good prognosis	0.5	-
Probability of poor prognosis	0.5	-
Maximum follow-up time	1.5 years	-
Impact of bad prognosis on survival	Log hazard ratio = 0.3	-
Survival time distribution	Weibull parameters:<br>Scale parameter 0.00001<br>Shape parameter 1.8	Weibull parameters in scenarios with high severity:<br>Scale parameter 0.00002<br>Shape parameter 1.8<br><br><u>Scenarios with moderate complexity of survivor function</u><br><br>Weibull parameters (low severity):<br>Mix 1: Scale parameter 0.00001<br>Shape parameter 2.0<br>Mix 2: Scale parameter 0.00001<br>Shape parameter 0.8<br>p = 0.5 (mix parameter)<br><br>Weibull parameters (high severity):<br>Mix 1: Scale parameter 0.00004<br>Shape parameter 2.0<br>Mix 2: Scale parameter 0.00004<br>Shape parameter 1.5<br>p = 0.5 (mix parameter)<br><br><u>Scenarios with high complexity of survivor function</u><br><br>Weibull parameters (low severity):<br>Mix 1: Scale parameter 0.00001<br>Shape parameter 2.0<br>Mix 2: Scale parameter 0.00001<br>Shape parameter 0.8<br>p = 0.5 (mix parameter)<br><br>Weibull parameters (high severity):<br>Mix 1: Scale parameter 0.00004<br>Shape parameter 2.0<br>Mix 2: Scale parameter 0.00004<br>Shape parameter 1.5<br>p = 0.5 (mix parameter)
Progression free survival	Overall survival time multiplied by a value from a beta distribution with shape parameters (5,10) – this implies the assumption that time to progression is 33% of OS. This is not an important assumption – time to progression is only included because we model a situation where switching cannot occur	-

	before disease progression	
Baseline treatment effect (note this is not the true treatment effect as this does not take into account the effect of the treatment that occurs through the time-dependent confounder, biomarker level, or the time-dependent part of the treatment effect, η)	Baseline log hazard ratio -0.22 for a small treatment effect	Alter log hazard ratio to -0.60 for a high treatment effect<br><br><u>Scenarios with moderate treatment effect time-dependency (moderately complex survivor function)</u><br><br>Baseline log hazard ratio in scenarios that include an additional time-dependent effect = -1.30<br><br>Alter log hazard ratio to -1.10 to maintain treatment effect with more severe disease<br><br>Alter log hazard ratio to -0.80 to represent a smaller treatment effect<br><br>Alter log hazard ratio to -0.65 to maintain smaller treatment effect with more severe disease<br><br><u>Scenarios with strong treatment effect time-dependency (highly complex survivor function)</u><br><br>Alter log hazard ratio to -2.20 for a high treatment effect<br><br>Alter log hazard ratio to -1.8 to maintain treatment effect with more severe disease<br><br>Alter log hazard ratio to -1.85 to represent a smaller treatment effect with moderate disease<br><br>Alter log hazard ratio to -1.35 to maintain smaller treatment effect with more severe disease
Biomarker intercept	Calculated using a normal distribution with mean of 20 and standard deviation of 1. Increased by 2.5 in patients who are in the poor prognosis group.	-
Biomarker value progression over time	As demonstrated by Equation (2). $\beta_2 = -0.02$ to represent that the biomarker value increases more slowly in the experimental group, and $\beta_1 = 0.04$ to indicate that the biomarker value increases over time	-
Impact of biomarker value on overall survival	As demonstrated by Equation (5). $\alpha = 0$ in scenarios with a constant treatment effect (i.e. those with a simple survivor function)	Increased biomarker value increases the risk of death. The strength of this relationship depends on the variable α, which equals 0.01 in scenarios with a moderate or complex survivor function.
Impact of biomarker value on treatment effect	$\alpha = 0$ in scenarios with a constant (zero time-dependency) treatment effect, hence the biomarker does not have an impact on the treatment effect	In scenarios with a moderately or highly complex survivor function treatment reduces the progression of the biomarker value and increased biomarker values increase the risk of death, hence the treatment has an additional effect through the biomarker. The strength of this relationship depends on the variable α, which equals

		0.01 in scenarios with a moderate or complex survivor function.<br><br>All scenarios with a moderate or complex survivor function include a time-dependent treatment effect in the experimental group. However, in selected scenarios the treatment effect received by switchers equals the average treatment effect in the experimental group, satisfying the 'common treatment effect' assumption.
Time-dependent portion of treatment effect, η	η =0 in scenarios with a constant (zero time-dependency) treatment effect	All scenarios with a moderate or complex survivor function include a time-dependent treatment effect in the experimental group. However, in selected scenarios the treatment effect received by switchers equals the average treatment effect in the experimental group, satisfying the 'common treatment effect' assumption<br><br>η =0.0025 to generate a reduction in the treatment effect over time in scenarios with a small treatment effect and a moderately complex survivor function/treatment effect time dependency.<br><br>η =0.003 to generate a reduction in the treatment effect over time in scenarios with a high treatment effect and a moderately complex survivor function/treatment effect time dependency.<br><br>η =0.006 to generate a reduction in the treatment effect over time in scenarios with a highly complex survivor function/stronger treatment effect time dependency
Assumed frequency of consultations	One every 3 weeks (21 days)	-
Probability of switching treatment over time	As shown in Table A1. This results in a switching proportion of approximately 24% in Scenario 1	Test a high switching scenario where all probabilities are increased – to an extent where approximately 50% of control group patients switch.
Prognosis of switching patients	As shown in Table A1. This makes switching more likely in good prognosis patients, via a mechanism that takes into account both time to progression and biomarker value at progression	As shown in Table A2. This makes switching more likely in poor prognosis patients, via a mechanism that takes into account both time to progression and biomarker value at progression
Treatment effect in switching patients	Equal to baseline treatment effect multiplied by ω. Set ω such that treatment effect received by switching patients is 80% of the average effect received by experimental group patients in base scenarios.	Alter ω such that the "common treatment effect" assumption holds – the treatment effect received by switching patients equals 100% of the average effect received by experimental group patients.

Appendix C: Scenario settings

Scenario	Switcher prognosis	Common treatment effect (CTE)	Treatment effect	Complexity of survivor function and time-dependency of treatment effect	Severity of disease	Switch proportion
1	Good	No CTE	Low	Simple / Zero	Low	Low
2	Poor	No CTE	Low	Simple / Zero	Low	Low
3	Good	CTE	Low	Simple / Zero	Low	Low
4	Poor	CTE	Low	Simple / Zero	Low	Low
5	Good	No CTE	High	Simple / Zero	Low	Low
6	Poor	No CTE	High	Simple / Zero	Low	Low
7	Good	CTE	High	Simple / Zero	Low	Low
8	Poor	CTE	High	Simple / Zero	Low	Low
9	Good	No CTE	Low	Mixture / Moderate	Low	Low
10	Poor	No CTE	Low	Mixture / Moderate	Low	Low
11	Good	CTE	Low	Mixture / Moderate	Low	Low
12	Poor	CTE	Low	Mixture / Moderate	Low	Low
13	Good	No CTE	High	Mixture / Moderate	Low	Low
14	Poor	No CTE	High	Mixture / Moderate	Low	Low
15	Good	CTE	High	Mixture / Moderate	Low	Low
16	Poor	CTE	High	Mixture / Moderate	Low	Low
17	Good	No CTE	Low	Mixture / Strong	Low	Low
18	Poor	No CTE	Low	Mixture / Strong	Low	Low
19	Good	CTE	Low	Mixture / Strong	Low	Low
20	Poor	CTE	Low	Mixture / Strong	Low	Low
21	Good	No CTE	High	Mixture / Strong	Low	Low
22	Poor	No CTE	High	Mixture / Strong	Low	Low
23	Good	CTE	High	Mixture / Strong	Low	Low
24	Poor	CTE	High	Mixture / Strong	Low	Low
25	Good	No CTE	Low	Simple / Zero	High	Low
26	Poor	No CTE	Low	Simple / Zero	High	Low
27	Good	CTE	Low	Simple / Zero	High	Low
28	Poor	CTE	Low	Simple / Zero	High	Low
29	Good	No CTE	High	Simple / Zero	High	Low
30	Poor	No CTE	High	Simple / Zero	High	Low
31	Good	CTE	High	Simple / Zero	High	Low
32	Poor	CTE	High	Simple / Zero	High	Low
33	Good	No CTE	Low	Mixture / Moderate	High	Low
34	Poor	No CTE	Low	Mixture / Moderate	High	Low
35	Good	CTE	Low	Mixture / Moderate	High	Low
36	Poor	CTE	Low	Mixture / Moderate	High	Low
37	Good	No CTE	High	Mixture / Moderate	High	Low
38	Poor	No CTE	High	Mixture / Moderate	High	Low
39	Good	CTE	High	Mixture / Moderate	High	Low
40	Poor	CTE	High	Mixture / Moderate	High	Low
41	Good	No CTE	Low	Mixture / Strong	High	Low
42	Poor	No CTE	Low	Mixture / Strong	High	Low
43	Good	CTE	Low	Mixture / Strong	High	Low
44	Poor	CTE	Low	Mixture / Strong	High	Low
45	Good	No CTE	High	Mixture / Strong	High	Low
46	Poor	No CTE	High	Mixture / Strong	High	Low
47	Good	CTE	High	Mixture / Strong	High	Low
48	Poor	CTE	High	Mixture / Strong	High	Low
49	Good	No CTE	Low	Simple / Zero	Low	Moderate
50	Poor	No CTE	Low	Simple / Zero	Low	Moderate
51	Good	CTE	Low	Simple / Zero	Low	Moderate
52	Poor	CTE	Low	Simple / Zero	Low	Moderate
53	Good	No CTE	High	Simple / Zero	Low	Moderate
54	Poor	No CTE	High	Simple / Zero	Low	Moderate
55	Good	CTE	High	Simple / Zero	Low	Moderate
56	Poor	CTE	High	Simple / Zero	Low	Moderate
57	Good	No CTE	Low	Mixture / Moderate	Low	Moderate
58	Poor	No CTE	Low	Mixture / Moderate	Low	Moderate

59	Good	CTE	Low	Mixture / Moderate	Low	Moderate
60	Poor	CTE	Low	Mixture / Moderate	Low	Moderate
61	Good	No CTE	High	Mixture / Moderate	Low	Moderate
62	Poor	No CTE	High	Mixture / Moderate	Low	Moderate
63	Good	CTE	High	Mixture / Moderate	Low	Moderate
64	Poor	CTE	High	Mixture / Moderate	Low	Moderate
65	Good	No CTE	Low	Mixture / Strong	Low	Moderate
66	Poor	No CTE	Low	Mixture / Strong	Low	Moderate
67	Good	CTE	Low	Mixture / Strong	Low	Moderate
68	Poor	CTE	Low	Mixture / Strong	Low	Moderate
69	Good	No CTE	High	Mixture / Strong	Low	Moderate
70	Poor	No CTE	High	Mixture / Strong	Low	Moderate
71	Good	CTE	High	Mixture / Strong	Low	Moderate
72	Poor	CTE	High	Mixture / Strong	Low	Moderate
73	Good	No CTE	Low	Simple / Zero	High	Moderate
74	Poor	No CTE	Low	Simple / Zero	High	Moderate
75	Good	CTE	Low	Simple / Zero	High	Moderate
76	Poor	CTE	Low	Simple / Zero	High	Moderate
77	Good	No CTE	High	Simple / Zero	High	Moderate
78	Poor	No CTE	High	Simple / Zero	High	Moderate
79	Good	CTE	High	Simple / Zero	High	Moderate
80	Poor	CTE	High	Simple / Zero	High	Moderate
81	Good	No CTE	Low	Mixture / Moderate	High	Moderate
82	Poor	No CTE	Low	Mixture / Moderate	High	Moderate
83	Good	CTE	Low	Mixture / Moderate	High	Moderate
84	Poor	CTE	Low	Mixture / Moderate	High	Moderate
85	Good	No CTE	High	Mixture / Moderate	High	Moderate
86	Poor	No CTE	High	Mixture / Moderate	High	Moderate
87	Good	CTE	High	Mixture / Moderate	High	Moderate
88	Poor	CTE	High	Mixture / Moderate	High	Moderate
89	Good	No CTE	Low	Mixture / Strong	High	Moderate
90	Poor	No CTE	Low	Mixture / Strong	High	Moderate
91	Good	CTE	Low	Mixture / Strong	High	Moderate
92	Poor	CTE	Low	Mixture / Strong	High	Moderate
93	Good	No CTE	High	Mixture / Strong	High	Moderate
94	Poor	No CTE	High	Mixture / Strong	High	Moderate
95	Good	CTE	High	Mixture / Strong	High	Moderate
96	Poor	CTE	High	Mixture / Strong	High	Moderate

Appendix D: Overview of simulation scenarios

Table D1 presents key details associated with each of the scenarios simulated. The true area under the curve (restricted mean survival time (RMST) at 548 days) unconfounded by treatment switching is presented, along with the average treatment effect in terms of a hazard ratio (HR) and an acceleration factor (AF). These were estimated by generating scenario data for 1,000,000 patients without applying switching – RMST was estimated directly from this data, Cox models were used to estimate the HR, and an RPSFTM under no switching was used to estimate the AF. The HR and AF represent only an approximation of the true treatment effect as the proportional hazards and constant acceleration factor assumptions do not hold. In terms of a hazard ratio, the average treatment effect varied between 0.54 and 0.81.

The proportion of control group patients that switched, averaged across the 1000 simulations that made up each scenario, is also presented. The switching proportion varied between 17% and 57% of all control group patients. Switching proportions are probabilistic and are reliant on other characteristics. Table D1 also presents the switching proportion as a percentage of the control group patients that became 'at-risk' of switching. In our simulations control group patients could only switch treatments if they were alive at their first 'consultation' at 21 days and if their disease progressed before the end of the simulated follow-up. The switching proportion as a percentage of patients that became at-risk of switching is higher than when it is measured as a percentage of all control group patients – it ranged from 21% to 58%. We estimated the proportion of patients who became at risk of switching in each scenario by collecting data on the number of patients for whom disease progression was observed in each simulation and taking the mean. This is approximate, but appropriately indicative for our purposes.

Table D1 also presents details on whether the treatment effect was assumed to be 'common' – that is, whether the treatment effect received by switchers was the same as the average treatment effect received by patients initially randomised to the experimental group. To provide further information on the strength of any violations in the common treatment effect assumption we also include details on the treatment effect size received by switchers. We also indicate the extent to which the treatment effect changed over time.

Table D1 also presents details on the mean proportion of patients that were censored in each scenario – that is, the proportion for whom death was not observed. This varied between 10% and 58%.

Table D1: Overview of simulated scenarios

Scenario	Switcher prognosis	Common treatment effect? (% of exp group treatment effect)	Treatment effect	Complexity of survivor function	Severity of disease	Switch proportion	Truth (years)		Average treatment effects		Treatment effect in switchers (AF)	Mean switcher % of total	Mean switcher % of at risk	Mean censoring proportion (%)
							RMST (Control group)	RMST (Exp group)	HR	AF				
1	Good	No CTE (80%)	Low	Simple	Low	Low	400.80	423.33	0.80	1.13	1.17	0.24	0.24	0.47
2	Poor	No CTE (80%)	Low	Simple	Low	Low	400.80	423.33	0.80	1.13	1.17	0.27	0.28	0.47
3	Good	CTE (100%)	Low	Simple	Low	Low	400.80	423.33	0.80	1.13	1.13	0.24	0.24	0.47
4	Poor	CTE (100%)	Low	Simple	Low	Low	400.80	423.33	0.80	1.13	1.13	0.27	0.28	0.47
5	Good	No CTE (80%)	High	Simple	Low	Low	400.80	456.60	0.55	1.40	1.43	0.24	0.24	0.56
6	Poor	No CTE (80%)	High	Simple	Low	Low	400.80	456.60	0.55	1.40	1.43	0.27	0.28	0.56
7	Good	CTE (100%)	High	Simple	Low	Low	400.80	456.60	0.55	1.40	1.40	0.24	0.24	0.56
8	Poor	CTE (100%)	High	Simple	Low	Low	400.80	456.60	0.55	1.40	1.40	0.27	0.28	0.56
9	Good	No CTE (80%)	Low	Moderate	Low	Low	357.46	391.12	0.81	1.19	1.15	0.17	0.25	0.40
10	Poor	No CTE (80%)	Low	Moderate	Low	Low	357.46	391.12	0.81	1.19	1.15	0.17	0.25	0.39
11	Good	CTE (100%)	Low	Moderate	Low	Low	357.46	391.12	0.81	1.19	1.19	0.17	0.25	0.40
12	Poor	CTE (100%)	Low	Moderate	Low	Low	357.46	391.12	0.81	1.19	1.19	0.17	0.24	0.39
13	Good	No CTE (80%)	High	Moderate	Low	Low	357.46	430.07	0.57	1.53	1.42	0.17	0.25	0.48
14	Poor	No CTE (80%)	High	Moderate	Low	Low	357.46	430.07	0.57	1.53	1.42	0.17	0.24	0.47
15	Good	CTE (100%)	High	Moderate	Low	Low	357.46	430.07	0.57	1.53	1.53	0.17	0.25	0.48
16	Poor	CTE (100%)	High	Moderate	Low	Low	357.46	430.07	0.57	1.53	1.53	0.17	0.24	0.47
17	Good	No CTE (80%)	Low	High	Low	Low	357.46	405.74	0.78	1.21	1.17	0.17	0.25	0.35
18	Poor	No CTE (80%)	Low	High	Low	Low	357.46	405.74	0.78	1.21	1.17	0.17	0.24	0.35
19	Good	CTE (100%)	Low	High	Low	Low	357.46	405.74	0.78	1.21	1.21	0.17	0.25	0.35
20	Poor	CTE (100%)	Low	High	Low	Low	357.46	405.74	0.78	1.21	1.21	0.17	0.24	0.35
21	Good	No CTE (80%)	High	High	Low	Low	357.46	438.20	0.56	1.52	1.41	0.17	0.25	0.44
22	Poor	No CTE (80%)	High	High	Low	Low	357.46	438.20	0.56	1.52	1.41	0.17	0.24	0.43
23	Good	CTE (100%)	High	High	Low	Low	357.46	438.20	0.56	1.52	1.52	0.17	0.25	0.44
24	Poor	CTE (100%)	High	High	Low	Low	357.46	438.20	0.56	1.52	1.52	0.17	0.25	0.43
25	Good	No CTE (80%)	Low	Simple	High	Low	315.41	343.95	0.80	1.13	1.20	0.24	0.25	0.23

Scenario	Switcher prognosis	Common treatment effect? (% of exp group treatment effect)	Treatment effect	Complexity of survivor function	Severity of disease	Switch proportion	Truth (years)		Average treatment effects		Treatment effect in switchers (AF)	Mean switcher % of total	Mean switcher % of at risk	Mean censoring proportion (%)
							RMST (Control group)	RMST (Exp group)	HR	AF				
26	Poor	No CTE (80%)	Low	Simple	High	Low	315.41	343.95	0.80	1.13	1.20	0.26	0.27	0.22
27	Good	CTE (100%)	Low	Simple	High	Low	315.41	343.95	0.80	1.13	1.13	0.24	0.25	0.23
28	Poor	CTE (100%)	Low	Simple	High	Low	315.41	343.95	0.80	1.13	1.13	0.26	0.26	0.22
29	Good	No CTE (80%)	High	Simple	High	Low	315.41	390.14	0.55	1.40	1.63	0.24	0.25	0.32
30	Poor	No CTE (80%)	High	Simple	High	Low	315.41	390.14	0.55	1.40	1.63	0.26	0.27	0.31
31	Good	CTE (100%)	High	Simple	High	Low	315.41	390.14	0.55	1.40	1.40	0.24	0.25	0.32
32	Poor	CTE (100%)	High	Simple	High	Low	315.41	390.14	0.55	1.40	1.40	0.26	0.26	0.31
33	Good	No CTE (80%)	Low	Moderate	High	Low	228.38	269.20	0.78	1.30	1.24	0.24	0.25	0.18
34	Poor	No CTE (80%)	Low	Moderate	High	Low	228.38	269.20	0.78	1.30	1.24	0.20	0.21	0.17
35	Good	CTE (100%)	Low	Moderate	High	Low	228.38	269.20	0.78	1.30	1.30	0.24	0.25	0.18
36	Poor	CTE (100%)	Low	Moderate	High	Low	228.38	269.20	0.78	1.30	1.30	0.20	0.21	0.17
37	Good	No CTE (80%)	High	Moderate	High	Low	228.38	322.24	0.56	1.85	1.68	0.24	0.25	0.25
38	Poor	No CTE (80%)	High	Moderate	High	Low	228.38	322.24	0.56	1.85	1.68	0.20	0.21	0.24
39	Good	CTE (100%)	High	Moderate	High	Low	228.38	322.24	0.56	1.85	1.85	0.24	0.25	0.25
40	Poor	CTE (100%)	High	Moderate	High	Low	228.38	322.24	0.56	1.85	1.85	0.20	0.21	0.24
41	Good	No CTE (80%)	Low	High	High	Low	228.38	273.39	0.81	1.23	1.19	0.24	0.25	0.11
42	Poor	No CTE (80%)	Low	High	High	Low	228.38	273.39	0.81	1.23	1.19	0.20	0.21	0.10
43	Good	CTE (100%)	Low	High	High	Low	228.38	273.39	0.81	1.23	1.23	0.24	0.25	0.11
44	Poor	CTE (100%)	Low	High	High	Low	228.38	273.39	0.81	1.23	1.23	0.20	0.21	0.10
45	Good	No CTE (80%)	High	High	High	Low	228.38	333.63	0.54	1.85	1.68	0.24	0.25	0.18
46	Poor	No CTE (80%)	High	High	High	Low	228.38	333.63	0.54	1.85	1.68	0.20	0.21	0.17
47	Good	CTE (100%)	High	High	High	Low	228.38	333.63	0.54	1.85	1.85	0.24	0.25	0.18
48	Poor	CTE (100%)	High	High	High	Low	228.38	333.63	0.54	1.85	1.85	0.20	0.21	0.18
49	Good	No CTE (80%)	Low	Simple	Low	Moderate	400.80	423.33	0.80	1.13	1.10	0.54	0.56	0.47
50	Poor	No CTE (80%)	Low	Simple	Low	Moderate	400.80	423.33	0.80	1.13	1.17	0.56	0.58	0.47
51	Good	CTE (100%)	Low	Simple	Low	Moderate	400.80	423.33	0.80	1.13	1.13	0.54	0.56	0.47

Scenario	Switcher prognosis	Common treatment effect? (% of exp group treatment effect)	Treatment effect	Complexity of survivor function	Severity of disease	Switch proportion	Truth (years)		Average treatment effects		Treatment effect in switchers (AF)	Mean switcher % of total	Mean switcher % of at risk	Mean censoring proportion (%)
							RMST (Control group)	RMST (Exp group)	HR	AF				
52	Poor	CTE (100%)	Low	Simple	Low	Moderate	400.80	423.33	0.80	1.13	1.13	0.56	0.58	0.48
53	Good	No CTE (80%)	High	Simple	Low	Moderate	400.80	456.60	0.55	1.40	1.32	0.53	0.55	0.57
54	Poor	No CTE (80%)	High	Simple	Low	Moderate	400.80	456.60	0.55	1.40	1.43	0.56	0.58	0.57
55	Good	CTE (100%)	High	Simple	Low	Moderate	400.80	456.60	0.55	1.40	1.40	0.53	0.55	0.57
56	Poor	CTE (100%)	High	Simple	Low	Moderate	400.80	456.60	0.55	1.40	1.40	0.56	0.58	0.58
57	Good	No CTE (80%)	Low	Moderate	Low	Moderate	357.46	391.12	0.81	1.19	1.15	0.40	0.57	0.40
58	Poor	No CTE (80%)	Low	Moderate	Low	Moderate	357.46	391.12	0.81	1.19	1.15	0.38	0.56	0.40
59	Good	CTE (100%)	Low	Moderate	Low	Moderate	357.46	391.12	0.81	1.19	1.19	0.39	0.57	0.40
60	Poor	CTE (100%)	Low	Moderate	Low	Moderate	357.46	391.12	0.81	1.19	1.19	0.39	0.56	0.40
61	Good	No CTE (80%)	High	Moderate	Low	Moderate	357.46	430.07	0.57	1.53	1.42	0.39	0.57	0.49
62	Poor	No CTE (80%)	High	Moderate	Low	Moderate	357.46	430.07	0.57	1.53	1.42	0.39	0.56	0.48
63	Good	CTE (100%)	High	Moderate	Low	Moderate	357.46	430.07	0.57	1.53	1.53	0.40	0.57	0.50
64	Poor	CTE (100%)	High	Moderate	Low	Moderate	357.46	430.07	0.57	1.53	1.53	0.38	0.56	0.48
65	Good	No CTE (80%)	Low	High	Low	Moderate	357.46	405.74	0.78	1.21	1.17	0.40	0.58	0.36
66	Poor	No CTE (80%)	Low	High	Low	Moderate	357.46	405.74	0.78	1.21	1.17	0.39	0.56	0.35
67	Good	CTE (100%)	Low	High	Low	Moderate	357.46	405.74	0.78	1.21	1.21	0.39	0.57	0.36
68	Poor	CTE (100%)	Low	High	Low	Moderate	357.46	405.74	0.78	1.21	1.21	0.39	0.56	0.35
69	Good	No CTE (80%)	High	High	Low	Moderate	357.46	438.20	0.56	1.52	1.41	0.40	0.57	0.45
70	Poor	No CTE (80%)	High	High	Low	Moderate	357.46	438.20	0.56	1.52	1.41	0.39	0.56	0.44
71	Good	CTE (100%)	High	High	Low	Moderate	357.46	438.20	0.56	1.52	1.41	0.40	0.57	0.46
72	Poor	CTE (100%)	High	High	Low	Moderate	357.46	438.20	0.56	1.52	1.52	0.38	0.56	0.44
73	Good	No CTE (80%)	Low	Simple	High	Moderate	315.41	343.95	0.80	1.13	1.10	0.55	0.56	0.23
74	Poor	No CTE (80%)	Low	Simple	High	Moderate	315.41	343.95	0.80	1.13	1.20	0.57	0.57	0.23
75	Good	CTE (100%)	Low	Simple	High	Moderate	315.41	343.95	0.80	1.13	1.13	0.56	0.56	0.23
76	Poor	CTE (100%)	Low	Simple	High	Moderate	315.41	343.95	0.80	1.13	1.13	0.57	0.57	0.23
77	Good	No CTE (80%)	High	Simple	High	Moderate	315.41	390.14	0.55	1.40	1.32	0.55	0.56	0.33

Scenario	Switcher prognosis	Common treatment effect? (% of exp group treatment effect)	Treatment effect	Complexity of survivor function	Severity of disease	Switch proportion	Truth (years)		Average treatment effects		Treatment effect in switchers (AF)	Mean switcher % of total	Mean switcher % of at risk	Mean censoring proportion (%)
							RMST (Control group)	RMST (Exp group)	HR	AF				
78	Poor	No CTE (80%)	High	Simple	High	Moderate	315.41	390.14	0.55	1.40	1.63	0.57	0.57	0.32
79	Good	CTE (100%)	High	Simple	High	Moderate	315.41	390.14	0.55	1.40	1.40	0.55	0.56	0.34
80	Poor	CTE (100%)	High	Simple	High	Moderate	315.41	390.14	0.55	1.40	1.40	0.57	0.57	0.33
81	Good	No CTE (80%)	Low	Moderate	High	Moderate	228.38	269.20	0.78	1.30	1.24	0.55	0.57	0.18
82	Poor	No CTE (80%)	Low	Moderate	High	Moderate	228.38	269.20	0.78	1.30	1.24	0.51	0.53	0.17
83	Good	CTE (100%)	Low	Moderate	High	Moderate	228.38	269.20	0.78	1.30	1.30	0.55	0.57	0.18
84	Poor	CTE (100%)	Low	Moderate	High	Moderate	228.38	269.20	0.78	1.30	1.30	0.51	0.53	0.17
85	Good	No CTE (80%)	High	Moderate	High	Moderate	228.38	322.24	0.56	1.85	1.68	0.55	0.58	0.26
86	Poor	No CTE (80%)	High	Moderate	High	Moderate	228.38	322.24	0.56	1.85	1.68	0.51	0.53	0.24
87	Good	CTE (100%)	High	Moderate	High	Moderate	228.38	322.24	0.56	1.85	1.85	0.55	0.57	0.26
88	Poor	CTE (100%)	High	Moderate	High	Moderate	228.38	322.24	0.56	1.85	1.85	0.51	0.53	0.24
89	Good	No CTE (80%)	Low	High	High	Moderate	228.38	273.39	0.81	1.23	1.19	0.55	0.58	0.11
90	Poor	No CTE (80%)	Low	High	High	Moderate	228.38	273.39	0.81	1.23	1.19	0.51	0.53	0.11
91	Good	CTE (100%)	Low	High	High	Moderate	228.38	273.39	0.81	1.23	1.23	0.55	0.58	0.11
92	Poor	CTE (100%)	Low	High	High	Moderate	228.38	273.39	0.81	1.23	1.23	0.51	0.53	0.11
93	Good	No CTE (80%)	High	High	High	Moderate	228.38	333.63	0.54	1.85	1.68	0.55	0.58	0.19
94	Poor	No CTE (80%)	High	High	High	Moderate	228.38	333.63	0.54	1.85	1.68	0.51	0.53	0.18
95	Good	CTE (100%)	High	High	High	Moderate	228.38	333.63	0.54	1.85	1.85	0.56	0.58	0.19
96	Poor	CTE (100%)	High	High	High	Moderate	228.38	333.63	0.54	1.85	1.85	0.51	0.53	0.18

Appendix E: Percentage bias across all scenarios (note different axis scales)

Figure E1: Percentage bias, Scenarios 1-24 (Low disease severity, low switch proportion)

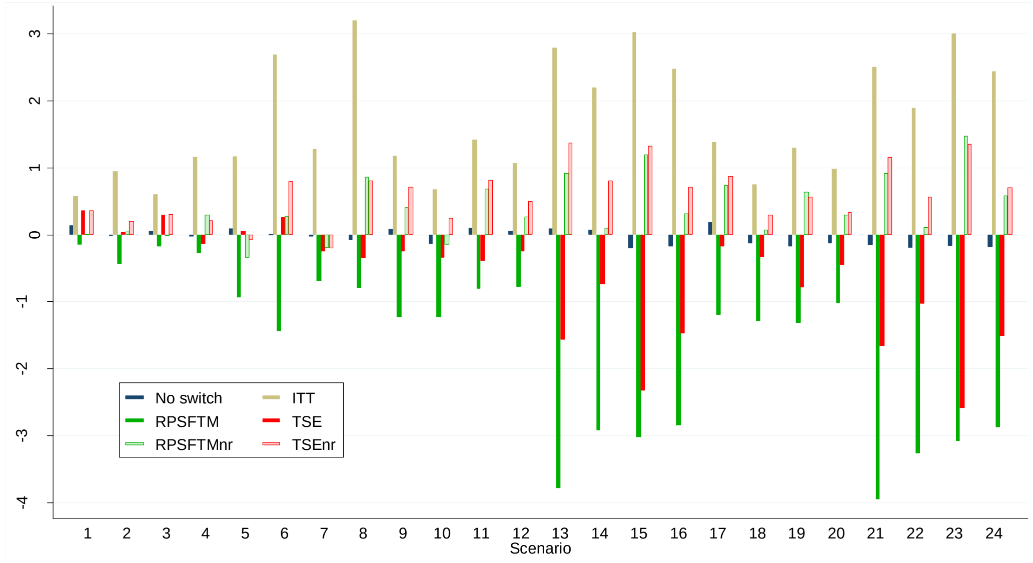

Figure E2: Percentage bias, Scenarios 49-72 (Low disease severity, moderate switch proportion)

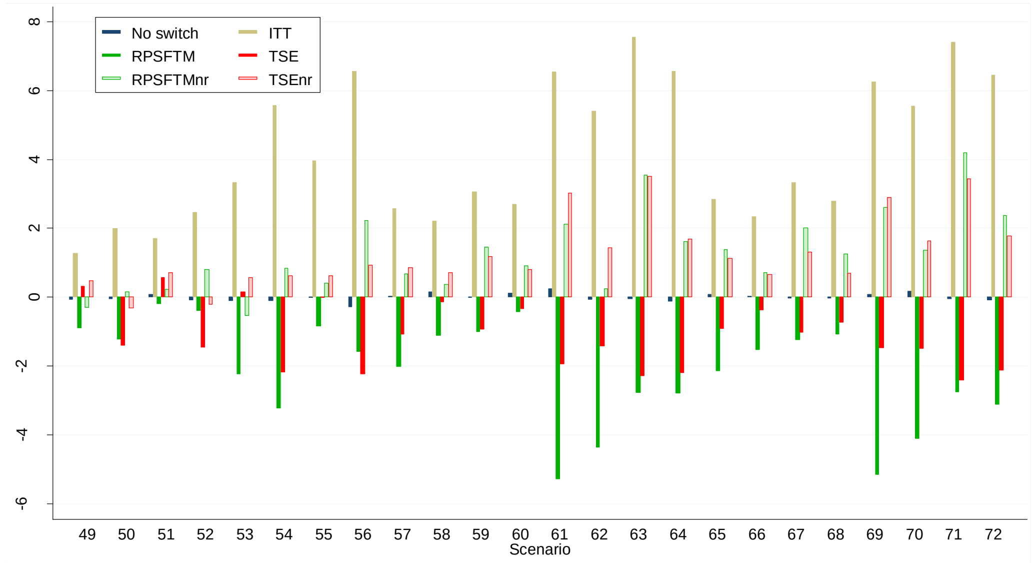

Figure E3: Percentage bias, Scenarios 25-48 (High disease severity, low switch proportion)

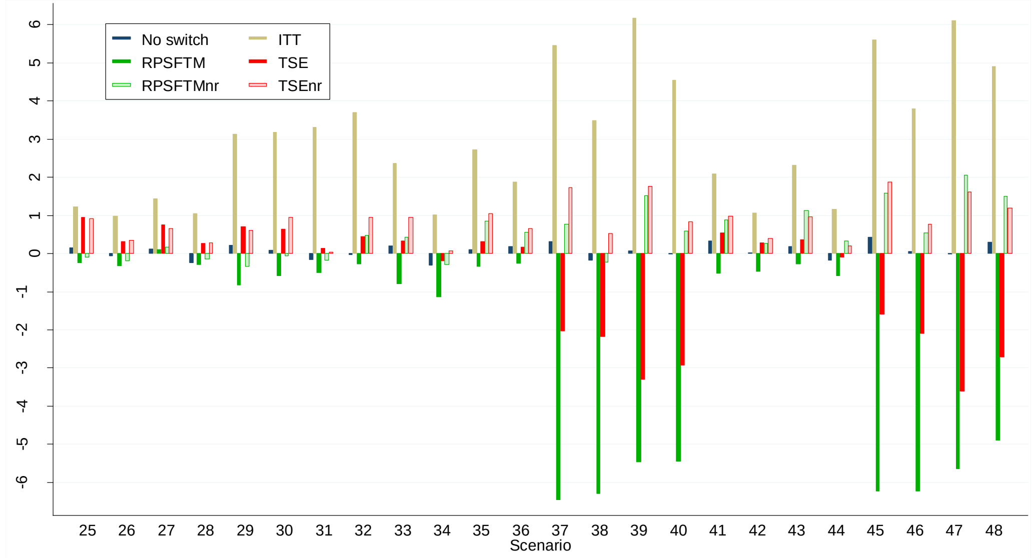

Figure E4: Percentage bias, Scenarios 73-96 (High disease severity, moderate switch proportion)

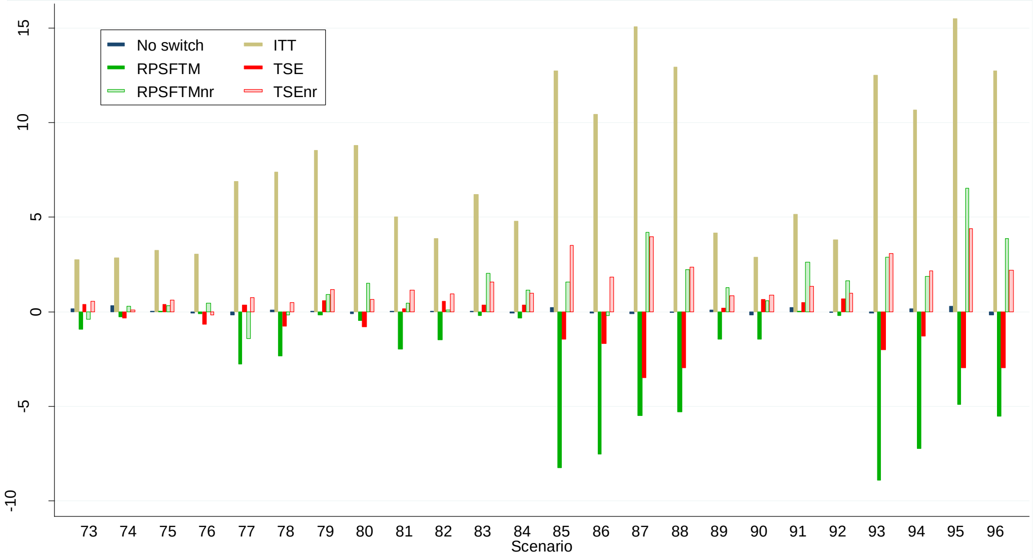

Appendix F: Empirical standard error of percentage bias across all scenarios (note different axis scales)

Figure F1: Empirical standard error, Scenarios 1-24 (Low disease severity, low switch proportion)

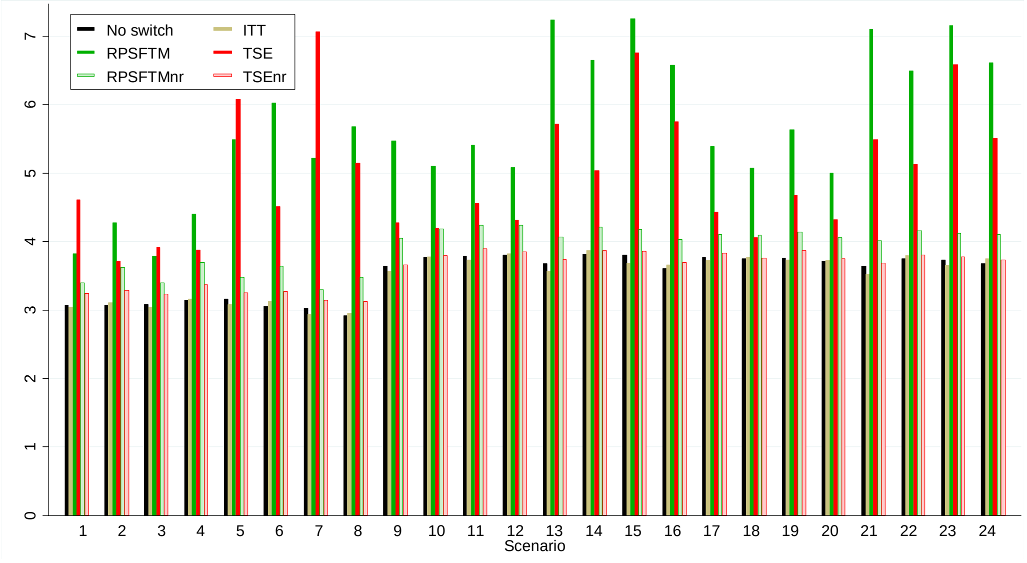

Figure F2: Empirical standard error, Scenarios 25-48 (Low disease severity, low switch proportion)

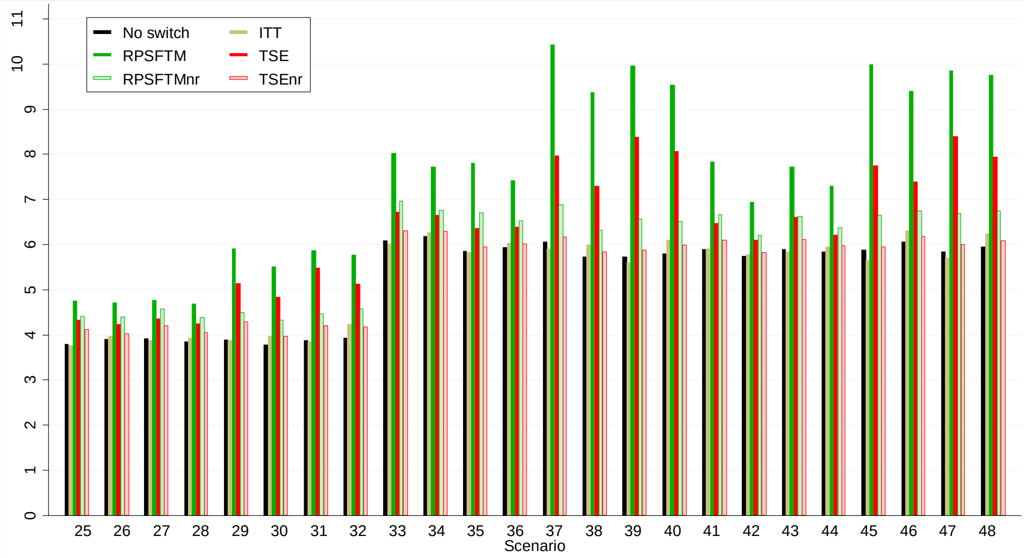

Figure F3: Empirical standard error, Scenarios 49-72 (Low disease severity, low switch proportion)

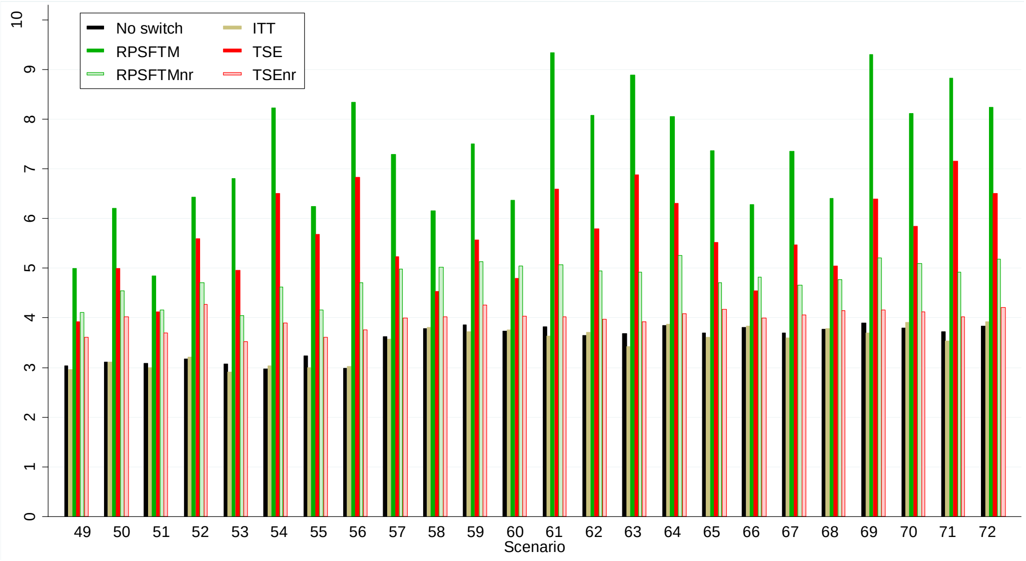

Figure F4: Empirical standard error, Scenarios 73-96 (Low disease severity, low switch proportion)

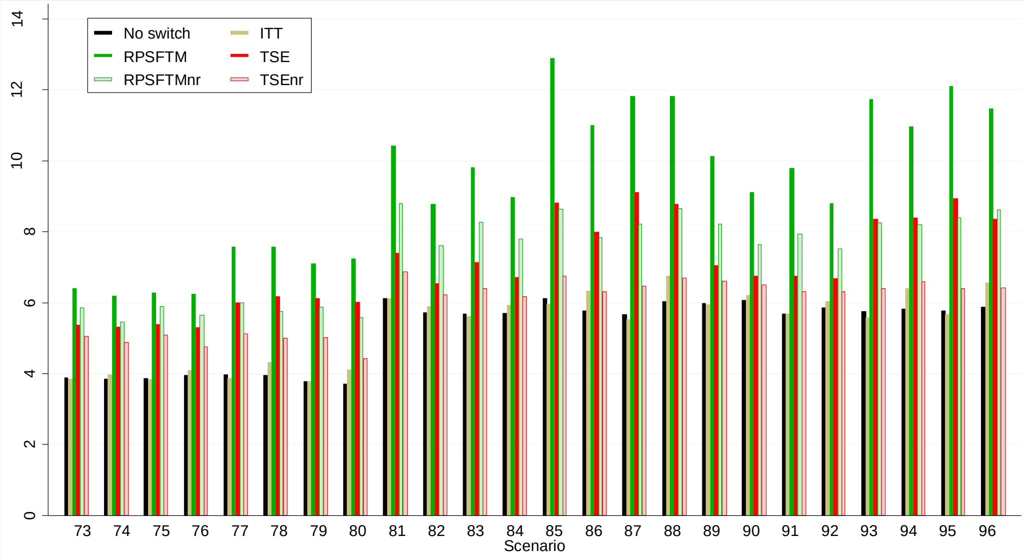

Appendix G: Root mean squared error of percentage bias across all scenarios (note different axis scales)

Figure G1: Root mean squared error, Scenarios 1-24 (Low disease severity, low switch proportion)

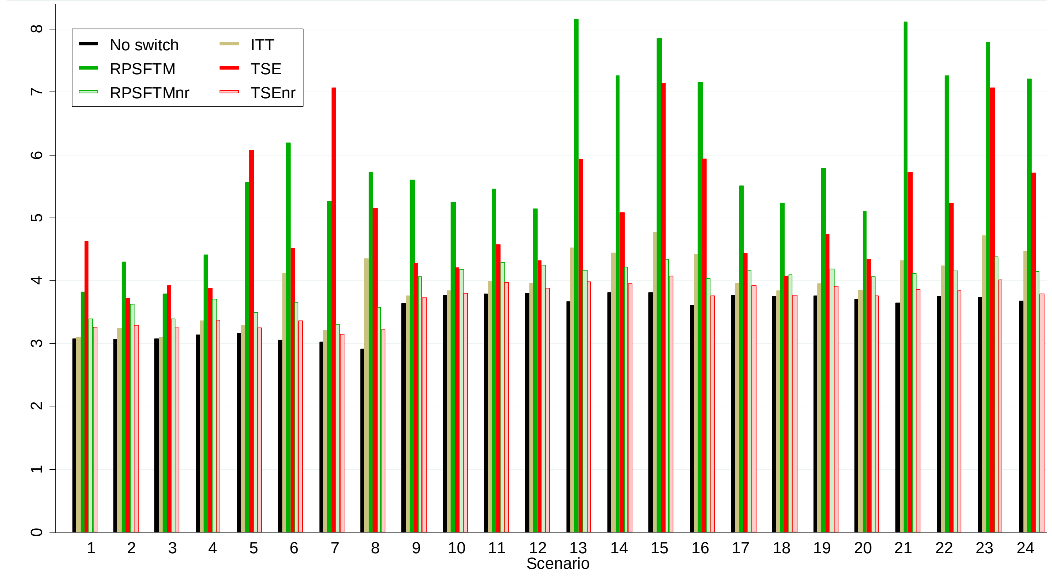

Figure G2: Root mean squared error, Scenarios 25-48 (Low disease severity, low switch proportion)

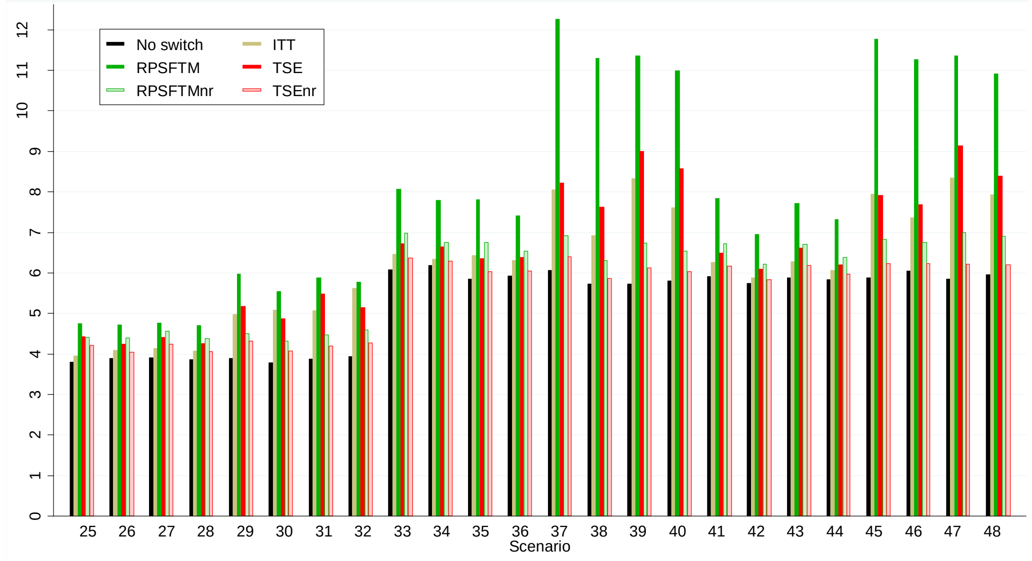

Figure G3: Root mean squared error, Scenarios 49-72 (Low disease severity, low switch proportion)

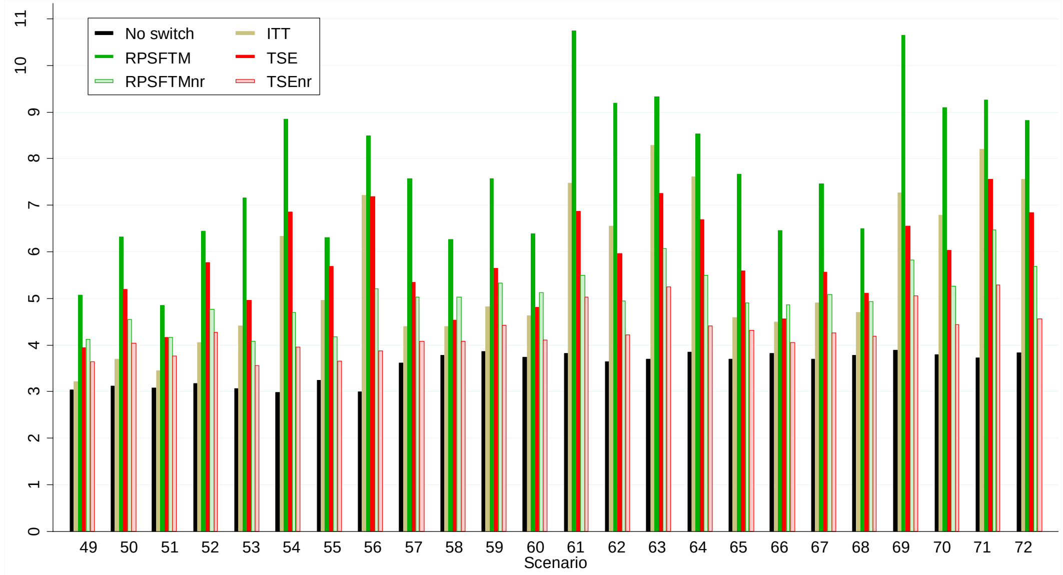

Figure G4: Root mean squared error, Scenarios 73-96 (Low disease severity, low switch proportion)

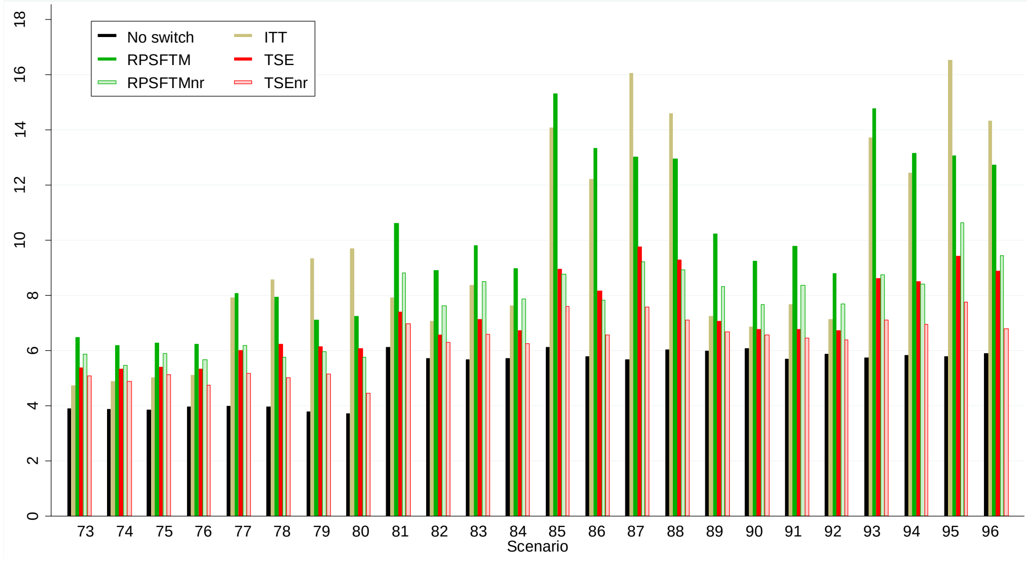

Appendix H: Further analysis of direction of bias associated with informative censoring

Our finding that the informative censoring bias associated with not re-censoring is likely to be associated with positive bias is important, and requires explanation. Our initial hypothesis was that despite our intentions, we had simulated switching in primarily poor prognosis patients even when we intended to simulate switching in primarily good prognosis patients. In the 32 scenarios in which there was a complex survivor function, a time-dependent treatment effect and a relatively low disease severity only approximately 69% of control group patients experienced disease progression and became eligible to switch – hence, even when switching was intended to be more likely in those with better prognosis, many of the best prognosis patients never became eligible to switch. Therefore, without re-censoring, positive informative censoring bias may be expected.

However, findings from scenarios in which there was a higher disease severity, and those in which there was not a complex survivor function and a time-dependent treatment effect, shed more light on this issue. In these scenarios, substantially more (approximately 96-98%) control group patients experienced disease progression and became eligible to switch treatments, and therefore there was a much clearer distinction in the prognosis of switchers and non-switchers. In some of these scenarios the directions of bias were more as might have been expected, For instance, in Scenarios 1, 5, 25, 29, 49, 53, 73 and 77 in which good prognosis patients were more likely to switch and the common treatment effect assumption did not hold, RPSFTMnr led to negative bias, and hence either the informative censoring led to negative bias (as would be expected), or the positive bias it created did not outweigh the negative bias associated with the violation of the common treatment effect assumption. However, in scenarios with a moderately complex survivor function, high disease severity and a violated common treatment effect assumption, the RPSFTMnr produced positive bias despite the clearer relationship between switch and prognosis and the negative bias expected due to violations of the common treatment effect assumption.

Closer inspection reveals that in these scenarios the RPSFTMnr did indeed over-estimate the treatment effect in switchers, but still resulted in positive bias in the estimate of mean survival – strongly indicating that the informative censoring led to positive bias. This finding was further supported in scenarios in which there was a constant treatment effect combined with a common treatment effect, in which we would expect to most clearly observe the impact of informative censoring because there are no competing biases (Scenarios 3, 4, 7, 27, 28, 31, 32, 51, 52, 55, 56, 75, 76, 79 and 80) – the RPSFTMnr continued to produce positive bias in the vast majority of

scenarios irrespective of the intended prognosis of switchers and despite the relatively clear relationship between prognosis and switch.

It is important to consider why non-re-censored analyses consistently increased estimates of control group mean survival compared to re-censored analyses. Non-re-censored analyses would be expected to lead to bias if participants who are censored at time *t* are not representative of all participants at risk in their randomised group at time *t*. Our analyses suggest that switchers who were not observed to die had worse prognosis than non-switchers at the censoring times that emerged after the estimation of counterfactual censoring times using RPSFTM and two-stage adjustment methods. This finding was apparent no matter whether patients with good or poor prognosis were more likely to switch treatments. This may appear surprising in scenarios where patients with good prognosis were more likely to switch. However, consider the situation when any non-switchers survive until the administrative censoring time-point – these patients demonstrably have good prognosis; they have long-term survival despite not receiving a beneficial treatment. Switchers, on the other hand, may have achieved long-term survival because they switched, not only because they had good prognosis. Even if switching was more likely to occur in patients with relatively good prognosis at the time of disease progression (when the switching decision was made), (not) re-censoring has its largest impact at much later time points at which point the prognostic balance between switchers and non-switchers may be substantially different due to the selection of patients that remain alive. At this point of the survival distribution, in situations where the experimental drug extends survival, it is plausible that non-switchers will generally have better prognosis than switchers.

White *et al.* (1999) suggest an empirical approach to demonstrate whether or not censoring is informative in the context of adjusting for treatment switching.[17] Informative censoring can be demonstrated if a variable can be found which predicts both censoring and event time. The authors suggest a plausible candidate to be a treatment history variable that indicates whether or not a patient has switched treatments. A series of time-dependent proportional hazards models can then be fitted, first with time-to-event as the outcome, and then with time-to-censoring as the outcome. Each model should be fitted on the original time scale, on the counterfactual time scale with re-censoring, and on the counterfactual time scale without re-censoring. According to White *et al.*, if the treatment history variable predicts events on all three time scales, but predicts censoring time only on the non-re-censored counterfactual time scale, this is consistent with censoring on the original scale being non-informative, and censoring on the counterfactual scale being informative without re-censoring and non-informative with re-censoring. We conducted this analysis on an example dataset from Scenario 85, in which

good prognosis patients were more likely to switch treatments, and indeed found that treatment history predicted time-to-event, but was predictive of censoring time only on the non-re-censored counterfactual scale.

Interestingly, in our analysis and in the analysis presented by White *et al.*, the hazard ratio estimated for the treatment history variable for time-to-event was substantially greater than 1.00, suggesting that a history of having switched treatments increased the hazard of death. In the analysis of White *et al.* it was stated that switchers had worse prognosis than non-switchers. However, in Scenario 85 of the simulation study presented here, better prognosis patients were more likely to switch, and 96% of control group patients experienced disease progression and hence became eligible to switch. This may be because in our simulated scenarios switching could only happen after progression, which is a strong predictor of death. Hence the risk of death after switching was actually higher than the risk of death in non-switchers at the same time-point because some of the non-switchers will not yet have progressed (and despite the fact that the treatment was beneficial and better prognosis patients were more likely to switch). In addition, as discussed previously, at late time-points non-switching long-term survivors may have generally better prognosis than survivors at the same time-point who had switched treatment. An estimated HR>1.00 is consistent with the non-re-censored analysis over-estimating survival: adjusting for switching leads switchers to have earlier censoring times leading to underestimation of the longer term risk of death in the control group.

Our finding that the informative censoring associated with not re-censoring is likely to result in positive bias is important, and is likely to be relevant in any study in which switching only becomes possible after a specified disease-related time-point, where a proportion of patients do not reach this time-point, and in any study where there are long-term survivors who do not switch.

Appendix I: Estimation of hazard ratios

Estimates of the treatment effect in terms of a hazard ratio were typically prone to much higher levels of percentage bias than estimates of restricted mean survival, particularly for methods that incorporated re-censoring when there was a time-dependent treatment effect. For instance, percentage bias in the average hazard ratio estimate of approximately 5% was often associated with very low (less than 1%) percentage bias in the estimate of restricted mean survival. This was particularly notable, in scenarios with a complex survivor function and a strong time-dependent treatment effect. In these scenarios TSE and RPSFTM methods that incorporated re-censoring often produced very high bias relating to estimates of the average hazard ratio (percentage bias usually in the region of 7-30%), but produced relatively low percentage bias with respect to restricted mean survival (often in the region of 1-4%). Methods that did not incorporate re-censoring were much less prone to substantial increases in percentage bias with respect to HR estimation compared to mean survival estimation when there was a time-dependent treatment effect – levels of percentage bias were high, but to a lesser extent.

Occasionally, in scenarios for which a particular method produced low levels of bias, the direction of bias was different for the estimate of the HR compared to the estimate of restricted mean survival. However, in the majority of cases the patterns and directions of bias associated with the different adjustment methods were similar when considering estimates of the average hazard ratio and estimates of restricted mean survival, though the levels of percentage bias were substantially higher when considering hazard ratios.

When the estimand is a treatment effect such as a HR, the main concern with applying re-censoring is whether or not the treatment effect changes over time. When the estimand is longer-term mean control group survival, the main concern is whether long-term control group hazards have been established within the timeframe of the re-censored dataset – which could be the case even if the treatment effect changes over time, or may not be the case even if the treatment effect remained constant over time. Whilst we found a relatively strong relationship between bias in RMST and bias in HRs in our study, this is likely to be due to the fact that our scenarios that had a time-dependent treatment effect also had a complex hazard function and those that had a constant treatment effect had a simplified hazard function – this is not guaranteed to be the case in reality.
